# Functional Materials Targeted Regulation of Gasdermins: From Fundamentals to Functionalities and Applications

**DOI:** 10.1002/advs.202500873

**Published:** 2025-03-24

**Authors:** Luyao Tian, Shuo Piao, Xia Li, Lanping Guo, Luqi Huang, Wenyuan Gao

**Affiliations:** ^1^ School of Pharmaceutical Science and Technology Tianjin University Tianjin 300072 P. R. China; ^2^ National Resource Center for Chinese Materia Medica China Academy of Chinese Medical Sciences Beijing 100700 P. R. China; ^3^ Key Laboratory of Pharmacology School of Pharmaceutical Science and Technology Tianjin University Tianjin 300072 P. R. China

**Keywords:** functionalities and applications, functional material, gasdermin, immune lanscape, pyroptosis

## Abstract

Targeted regulation of pyroptosis to modulate the immune landscape has emerged as a novel design strategy for cancer immunotherapy and anti‐inflammatory therapy. However, pyroptosis acts as a double‐edged sword, making it important to optimize the design strategies of functional materials to appropriately activate pyroptosis for effective disease treatment. This paper summarizes and discusses the structure, pore formation, and molecular mechanisms of “executor” Gasdermins, as well as the events preceding and following these processes. Subsequently, the focus is on reviewing functional materials that directly regulate Gasdermin pore formation to target pyroptosis and those that indirectly regulate the events before and after Gasdermin pore formation to control pyroptosis activity. Finally, the advantages, disadvantages, and future prospects of designing such functional materials are provided, aiming to facilitate the precise design, pharmacological investigation, and clinical translation of pyroptosis‐related functional materials.

## Graphical abstract

Targeted regulation of pyroptosis to modulate the immune landscape is a novel design strategy for cancer immunotherapy and anti‐inflammatory treatment. Pyroptosis, as a double‐edged sword, underscores the importance of optimizing the design strategy of functional materials and appropriately activating pyroptosis for the effective treatment of diseases.

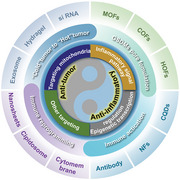

## 1 Introduction

**Figure 1:**
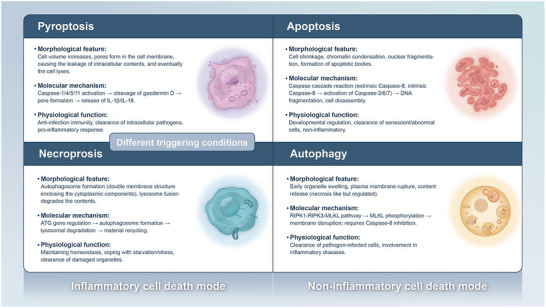
Comparison chart of pyroptosis, apoptosis, autophagy, and necroptosis, creating artboards in Adobe Illustrator.

**Figure 2:**
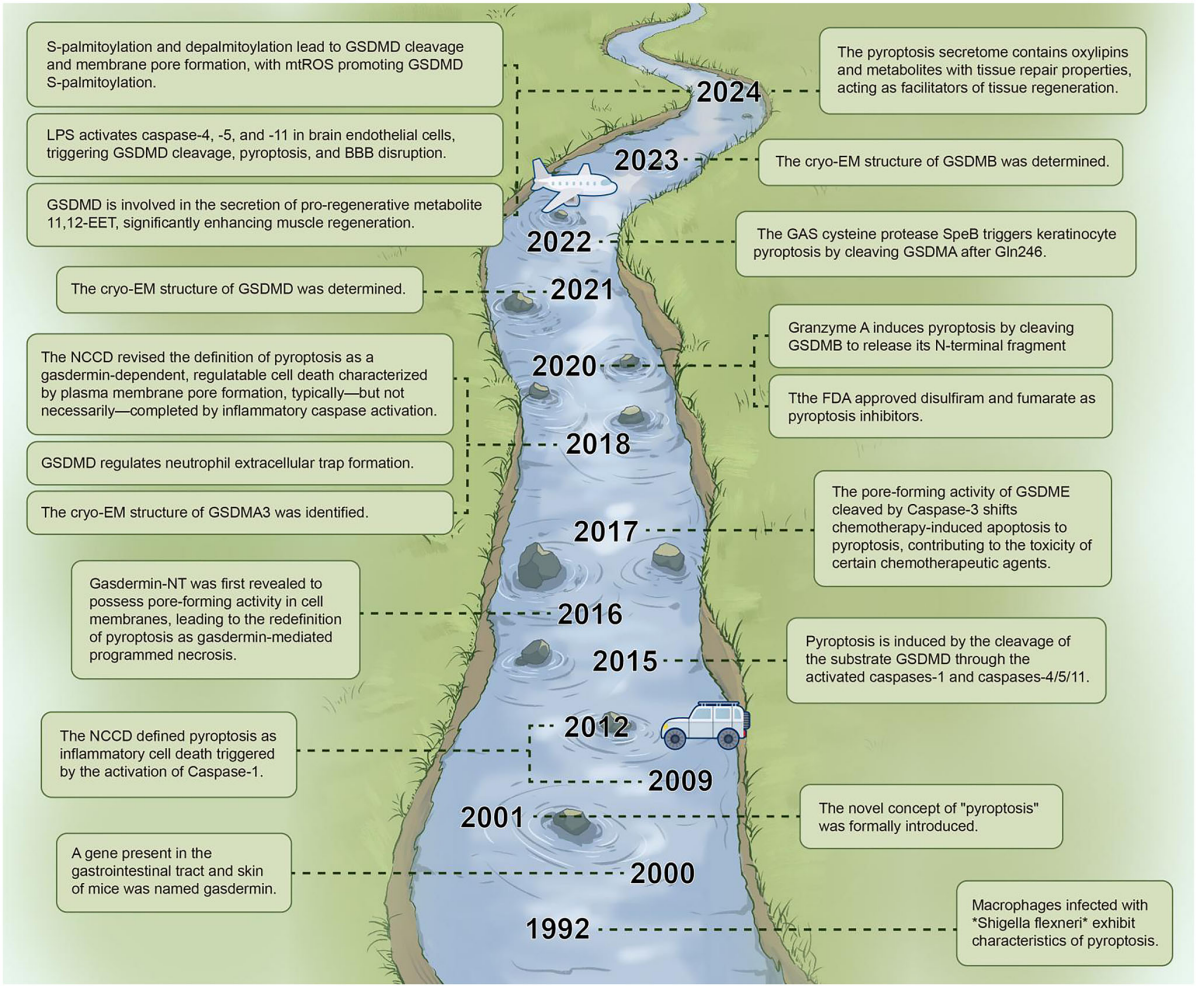
Pyroptosis and gasdermins, creating artboards in Adobe Illustrator.

Pyroptosis is one of the inflammatory forms of programmed cell death caused by the gasdermins,^[^ ^1^ ^]^ primarily characterized by cell swelling and pore formation in the plasma membrane, leading to cell lysis and the release of cellular contents.^[^ ^2^, ^3^, ^4^ ^]^ This process triggers an inflammatory response, activating the innate or adaptive immune systems and eliciting a strong immune reaction.^[^ ^2^, ^3^, ^4^ ^]^ As shown in **Figure** **1**, **2**, in contrast, apoptosis and autophagy are generally considered as “quiet cell death” modalities, characterized by the relative integrity of the cell membrane, and play crucial roles in maintaining intracellular homeostasis and tissue function.^[^ ^5^ ^]^ Furthermore, the molecular mechanisms of apoptosis and autophagy primarily involve the regulation of intracellular signaling pathways, whereas pyroptosis is closely associated with inflammasome activation and the Gasdermin family of proteins. Regarding the biological functions of pyroptosis in the body, recent studies have demonstrated that pyroptosis acts as a “double‐edged sword” from multiple perspectives.^[^ ^6^, ^7^ ^]^ On the positive hand, pyroptosis can induce the immune response, maintain internal homeostasis, activate anti‐tumor immunity, and synergize with immune checkpoints to convert “cold tumors” into “hot tumors,” collectively promoting the activation of the immune system.^[^ ^8^ ^]^ On the other hand, it should be noted that long‐term chronic inflammation from pyroptosis can promote cancer development.^[^ ^9^, ^10^, ^11^ ^]^ In addition, acute activation of pyroptosis can lead to the infiltration of the immune system, thereby inhibiting tumor growth.^[^ ^12^ ^]^ But it is crucial to point out that excessive activation of the immune system can result in an overactive inflammatory response, unexpected cell death, tissue damage, and ultimately “cytokine storms,” harmful inflammation, or even organ dysfunction.^[^ ^13^, ^14^, ^15^, ^16^ ^]^ Therefore, an increasing number of studies are focusing on harnessing the positive effects of pyroptosis while simultaneously minimizing side effects, aiming to utilize pyroptosis for disease treatment.″

The formation of Gasdermin pores is a necessary step in the activation of pyroptosis, and as “executors” gasdermins can function to target the regulation of pyroptosis and gulating pore formation.^[^ ^4^, ^17^, ^18^ ^]^ The Gasdermin family is a group of proteins with sequence homology that has gained considerable recognition in recent years due to its essential role as an “executor” in pyroptosis.^[^ ^19^ ^]^ Under normal physiological conditions, the C‐terminal domain of Gasdermins inhibits the pyroptotic activity of the N‐terminal domain.^[^ ^20^ ^]^ However, upon receiving stimulation signals, inflammasomes cleave Gasdermins, releasing the N‐terminal fragment, which forms circular pore‐like channels of varying sizes in the plasma membrane. This leads to the release of cellular contents, mainly including HMGB1, ATP, IL‐18, and IL‐1β, causing local inflammatory responses and immune responses. These processes are closely related to inflammatory diseases and cancer.^[^ ^19^ ^]^ When applied to cancer drugs, it can enhance antitumor immune responses. In the design of anti‐inflammatory drugs, the degree of pyroptosis can be inhibited to reduce local inflammation.

Previous studies investigated natural functional materials, such as Quercetin, Curcumin, Betaine, etc., highlighting the potential of these materials in regulating pyroptosis signaling pathways for the treatment of cancer and inflammatory diseases.^[^ ^21^, ^22^, ^23^, ^24^, ^25^, ^26^, ^27^, ^28^, ^29^, ^30^, ^31^, ^32^, ^33^, ^34^, ^35^, ^36^, ^37^, ^38^, ^39^, ^40^ ^]^ Besides that, Intelligent composite materials, with therapeutic strategies such as Photodynamic Therapy (PDT), Photothermal Therapy (PTT), Ion‐Interference Synergistic Therapies, Radiation Therapy, and Ultrasound Therapy, have also shown research prospects in inducing pyroptosis in tumor cells and combating inflammatory microenvironments.^[^ ^41^, ^42^, ^43^, ^44^, ^45^, ^46^, ^47^, ^48^, ^49^, ^50^, ^51^, ^52^, ^53^, ^54^, ^55^, ^56^, ^57^, ^58^, ^59^, ^60^, ^61^, ^62^, ^63^, ^64^, ^65^, ^66^, ^67^, ^68^, ^69^, ^70^, ^71^, ^72^, ^73^, ^74^, ^75^, ^76^, ^77^, ^78^, ^79^, ^80^ ^]^

The continuous emergence of functional materials aimed at the targeted regulation of pyroptosis, coupled with diverse synergistic strategies, necessitates a comprehensive review of how these materials can appropriately modulate pyroptosis pathways. At present, most researchers concentrate on elucidating the application of functional materials in regulating pyroptosis signaling pathways from a materials science perspective for the treatment of cancer. However, we observed that the molecular mechanism features of pyroptosis can apply to functional materials’ strategy. Therefore, we consider gasdermin pore formation as the central event, dividing the immune activation signals upon Gasdermin pore formation and the changes in the immune landscape after pore formation into three parts to explain pyroptosis from another perspective. In this review, we first provide an overview of the interplay between pyroptosis and Gasdermins pore formation. We then summarize and discuss the structure of Gasdermins, pore formation, and the molecular events before and after pore formation. For precise disease treatment, the biological characteristics of Gasdermins can be utilized in the strategic design of functional materials, with the aim of providing new ideas for design and optimization for researchers engaged in the development of such functional materials. Subsequently, we focus on reviewing functional materials that directly regulate Gasdermin pore formation based on pyroptosis modulation, as well as those that indirectly regulate the molecular events surrounding Gasdermin pore formation. Finally, we discuss the advantages, disadvantages, and future prospects of designing such functional materials. We believe that functional materials targeting the regulation of Gasdermins hold significant promise for widespread application and clinical translation in tumor immunotherapy and anti‐inflammatory therapy.

## 2 Gasdermins: Executors of Pyroptosis

As shown in Figure 1, as early as 1992, macrophages infected with Shigella flexneri were observed by researchers to exhibit characteristics of pyroptotic cell death.^[^ ^3^ ^]^ However, for a long period, this form of cell death was mistakenly thought to be apoptosis. It was not until 2001 that the novel concept of pyroptosis entered the scientific community's awareness. Initially, the Nomenclature Committee on Cell Death (NCCD) twice defined pyroptosis as inflammatory cell death induced by Caspase‐1 activation.

In a 2015 study, it was discovered that the Gasdermin family member GSDMD is a key protein in the pyroptosis pathway mediated by Caspase‐1/4/5/11. GSDMD can specifically cleave the linker between the N‐terminal Gasdermin‐NT and the C‐terminal Gasdermin‐CT, demonstrating that Gasdermin‐NT has pyroptotic activity.^[^ ^81^, ^82^ ^]^ The naming of Gasdermins is based on the expression sites of one of its subtypes, GSDMA, derived from “gastric” and “dermatological.”^[^ ^83^ ^]^ Other family members have been identified due to their homology with DFNA5, including GSDMA, GSDMB, GSDMC, GSDMD, GSDME (DFNA5), and GSDMF (PJVK/DFNB59).

Since 2015, research into the mechanisms of the Gasdermin family has deepened, revealing that inflammatory Caspases can induce pyroptosis through GSDMD. This work also uncovered for the first time that GSDM‐NT has the ability to perforate cell membranes, thereby destructing them, redefining pyroptosis as cell programmed necrosis mediated by Gasdermins.^[^ ^4^ ^]^ In 2017, GSDME was found to have a function similar to GSDMD; specifically, Caspase‐3 can cleave and activate GSDME, leading it to form pores in the membrane and trigger pyroptosis. In the same year, it was identified as a Caspase‐3 substrate capable of converting apoptosis into pyroptosis.^[^ ^84^, ^85^ ^]^ Subsequently, it was discovered that pyroptosis can be triggered not only by Caspases but also by Granzyme A, which induces pyroptosis by cleaving GSDMB to release its N‐terminal fragment.^[^ ^86^ ^]^ The NCCD revised the definition of pyroptosis to: a regulated cell death dependent on the formation of plasma membrane pores by Gasdermins, often but not always accomplished through the activation of inflammatory Caspases.^[^ ^86^ ^]^ In 2022, the SpeB virulence factor from the GAS cysteine protease was found to trigger keratinocyte pyroptosis by cleaving GSDMA after Gln246, releasing an active N‐terminal fragment that can enhance the host's ability to recognize and control the virulence of dangerous microbial pathogens via a simple single‐molecule mechanism.^[^ ^87^ ^]^ Recently, it has been discovered that cleavage is not the sole activator of GSDMD. Mitochondria‐generated reactive oxygen species (ROS) can promote the S‐palmitoylation of GSDMD, and both S‐palmitoylation and depalmitoylation can lead to GSDMD cleavage and membrane pore formation. Furthermore, reversible S‐palmitoylation serves as a universal switch for the activation of the entire Gasdermins.^[^ ^88^ ^]^

In 2018, the first cryo‐electron microscopy structure of Gasdermins, GSDMA3, was published. Subsequently, the cryo‐EM structures of GSDMD and GSDMB were also identified.^[^ ^89^, ^90^ ^]^ These studies revealed the mechanism of pore formation by GSDMD and its role as a conduit for IL‐1 secretion, laying the foundation for further research into the activity of various Gasdermins. The structural differences among the subtypes can also provide insights for the development of immunotherapies.

Additionally, in recent years, the FDA has approved two pyroptosis inhibitors: disulfiram and fumarate.^[^ ^91^, ^92^ ^]^ Both work by modifying the cysteine residues of Gasdermins (GSDMs) to prevent their oligomerization and pore formation, providing a foundation and reference for the development of future pyroptosis regulators.

The pyroptosis pathway is complex and not yet fully elucidated; however, pore formation by Gasdermins is a critical and indispensable process. To facilitate the summary and analysis of the pyroptosis pathway's mechanisms, we categorize pyroptosis into pre‐pore formation events, pore formation, and post‐pore formation responses under physiological and pathological conditions. This categorization aims to provide a reference for simplifying the development of functional materials.

## 3 The Core Event of Pyroptosis: Gasdermin Pores

### 3.1 Gasdermin Pore Formation: Pyroptosis

#### 3.1.1 Structure

**Figure 3:**
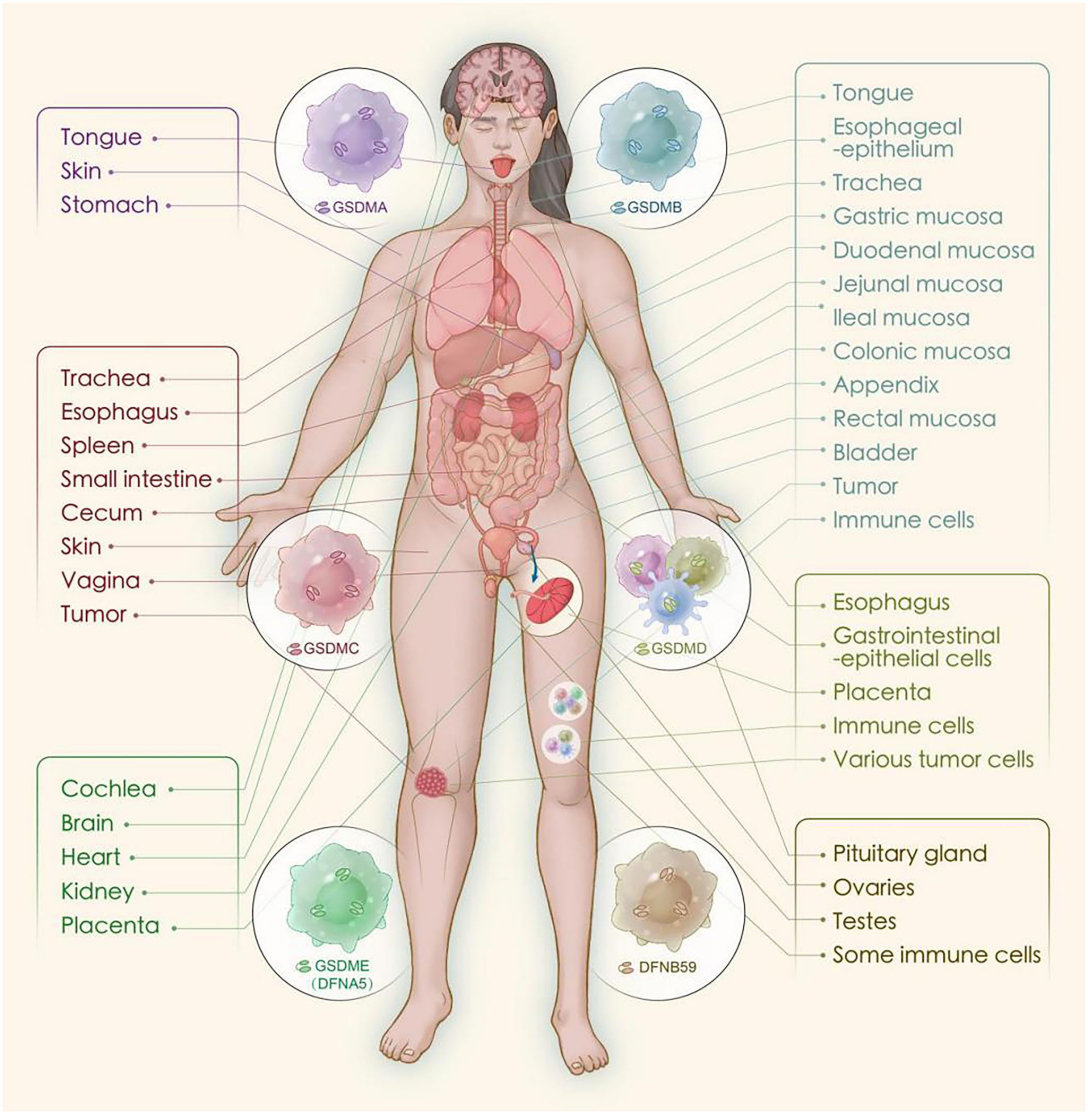
Gasdermins expression location, creating artboards in Adobe Illustrator.

Gasdermins are a family of proteins with sequence homology. Although their expression sites vary, their structures are highly similar, and their most important function, pyroptotic activity, is closely related to their structure. As mentioned earlier, they consist of six subtypes, all of which, except for GSDMF, are composed of a C‐terminal repressor domain, a cytotoxic N‐terminal domain, and a flexible linker domain.^[^ ^93^ ^]^ Early research has found that PJVK homologous genes exist in early vertebrates and invertebrates, leading to the speculation that Gasdermins may have evolved from these ancestors.^[^ ^94^ ^]^ As shown in **Figure** **3**, based on their abundance in tumors, we categorize Gasdermins into two groups: those that are highly expressed in tumors and those that are lowly expressed in tumors.

##### Gasdermins with High Abundance in Tumors

The design strategies for proteins with high abundance in tumors are distinct from those with low expression levels; typically, inducing direct pore formation is sufficient for the former. In contrast, when it comes to anti‐inflammatory design strategies, the approaches for both high‐ and low‐expressing proteins converge, focusing on either directly or indirectly inhibiting the pore‐forming activity of these proteins. Among the proteins that are highly expressed in tumors, GSDMB, GSDMC, and GSDMD are the primary examples.

In humans, GSDMB is located at the chromosomal locus 17q21.1, producing at least six transcripts that are translated into four isoforms, namely GSDMB1, GSDMB2, GSDMB3, and GSDMB4 (NCBI Gene ID: 55876).^[^ ^95^ ^]^ However, it is important to note that there is no GSDMB ortholog in mice, which makes studying the physiological and pathological roles of GSDMB in animal models challenging. This also indicates that GSDMB is a functional gene that has evolved in humans and other mammals.^[^ ^96^, ^97^ ^]^

GSDMB is primarily abundant in the esophageal epithelium, tongue, gastric mucosa, duodenal mucosa, jejunal mucosa, ileal mucosa, appendix, colonic mucosa, rectal mucosa, trachea, and bladder in humans; it is highly expressed in some esophageal, gastric, intestinal, and ovarian cancer cell lines; in human clinical tumor samples, GSDMB is commonly expressed in colorectal cancer, rectal cancer, pancreatic cancer, and cervical cancer, but its expression in normal tissues is higher than that in human clinical tumor samples; however, it is rarely or not expressed in liver, breast, or lung cancer samples, indicating that GSDMB is selectively absent in advanced cancers.^[^ ^86^, ^98^, ^99^ ^]^ In ≈60% of HER2‐positive breast cancers, the GSDMB protein is overexpressed, and it is often co‐expressed with the oncogene HER2/Erbb2 in clinical sample studies.^[^ ^100^, ^101^ ^]^ Unprocessed GSDMB has the role of promoting tumor occurrence, development, and drug resistance.^[^ ^100^, ^102^, ^103^ ^]^

It is worth noting that some studies suggest that the activation of GSDMB does not lead to cell lysis but instead causes the lysis of intracellular bacteria, thereby playing an antibacterial role. However, this process can be disrupted by the Shigella flexneri, an invasive enteric pathogen, which secretes the type III effector protein IpaH7.8 to ubiquitinate GSDMB, leading to its degradation by the 26S proteasome, thus sparking a period of controversy regarding the pyroptotic capability of GSDMB.^[^ ^97^ ^]^ It was not until later that the specific mechanism of its pyroptotic activity was proven. Previously, it was believed that GSDMB lacked self‐inhibition and exhibited clear lipid‐binding ability in its full‐length form, which is why there has always been less research on GSDMB.^[^ ^104^ ^]^ However, in later studies, it was found that the full‐length GSDMB has stronger self‐inhibition, and there was a period when the pyroptotic activity of GSDMB was questioned, only to later discover that it is the nonfunctional isoforms with missing or modified exon 6 that determine whether different subtypes of GSDMB have pyroptotic activity. Only isoforms with an intact exon 6 possess the ability to induce pyroptosis. Since different cancer cell lines are composed of various distinct isoforms, this also explains the recent differing opinions on the pyroptotic capability of GSDMB. Studies have shown that the isoforms of GSDMB that have pyroptotic effects are only GSDMB3 and GSDMB4 (partial pyroptosis, partial apoptosis), with pyroptosis also being accompanied by the mitochondrial damage common to pyroptosis.^[^ ^95^, ^105^, ^106^, ^107^ ^]^

Studies have indicated that the homozygous genotype CC of GSDMB has a protective effect against allergic rhinitis.^[^ ^108^ ^]^ Genetic research has demonstrated a correlation between the polymorphisms (SNPs) of GSDMB and an increased susceptibility to complex trait inflammatory diseases, such as Crohn's disease, ulcerative colitis, and asthma.^[^ ^109^, ^110^ ^]^ Therefore, GSDMB holds promise as a therapeutic target for the aforementioned conditions. Additionally, the cytokine interferon‐γ has been proven to induce the upregulation of GSDMB expression, thereby triggering immune responses.^[^ ^86^ ^]^

GSDMC is located at the 8q24.21 region on human chromosome 8. In the mouse genome, GSDMC has four isoforms, namely mGSDMC1, mGSDMC2, mGSDMC3, and mGSDMC4. GSDMC and GSDMC1 were first identified as biomarkers for melanoma and were later identified as members of the Gasdermins family based on their sequence homology. They are also the only members whose biological functions have not yet been determined.^[^ ^94^, ^111^, ^112^, ^113^ ^]^

GSDMD is the most extensively and deeply studied member of the Gasdermins family and was the first protein in which pyroptotic activity was discovered among Gasdermins. GSDMD is primarily expressed in immune cells, predominantly macrophages and dendritic cells, as well as in the placenta, esophageal and gastrointestinal epithelial cells, and various tumor cells.^[^ ^98^, ^114^, ^115^, ^116^ ^]^ In the human genome, GSDMD is located at the 8q24.3 locus on chromosome 8, with a full‐length molecular weight of 53kDa, of which 31kDa is the N‐terminal domain, and the interface between the N‐terminus and C‐terminus maintains it in an auto‐inhibited conformation.^[^ ^117^ ^]^ Initially, it was identified due to its homology with other GSDM genes near GSDMC on chromosome 8.^[^ ^114^ ^]^

The four basic residues of a pair of amphipathic α‐helices in GSDMD‐NT are responsible for protein oligomerization and pyroptosis.^[^ ^118^, ^119^ ^]^ When cleaved, the N‐terminus can expose basic amino acid residues, and studies have shown that they can interact with acidic lipids (such as phosphatidylinositol phosphates, phosphatidylserine, phosphatidic acid, and cardiolipin), leading to binding with target membranes.^[^ ^89^ ^]^ However, it does not bind with phosphatidylethanolamine (PE) or phosphatidylcholine (PC), which are the major lipids in the plasma membrane leaflet. It is worth noting that the outer leaflet of endosomal and phagosomal membranes contains the same phospholipids as the inner leaflet of the plasma membrane, indicating that GSDMD‐NT may also bind to these organelles. GSDMD‐NT does not harm bystander cells; that is, it only binds to phospholipids on the inner leaflet of the plasma membrane of living cells, so it does not disrupt the plasma membrane from the outside.^[^ ^118^ ^]^ At the same time, the exposure of phosphatidylserine after pyroptosis can enhance the activation of the coagulation initiator tissue factor TF.^[^ ^120^ ^]^ Additionally, studies have presented the crystal structure of the complex formed by human CASP1 and full‐length mouse GSDMD, elucidating that the loop structure of the GSDMD linker binds to the active site of caspase‐1, and the hydrophobic pocket of the C‐terminus of GSDMD binds to the caspase exon. This has led to the proposal of other pathways targeting Gasdermins.^[^ ^121^, ^122^ ^]^

##### Gasdermins with Low Abundance in Tumors

Strategies for applying proteins with low abundance in tumors to pyroptosis involve not only inducing pore formation through various pathways but also enhancing the expression of intracellular response proteins. For instance, GSDME is underexpressed in tumors due to high promoter methylation. Therefore, DNA methyltransferases can be introduced into nanomedicines to increase its expression before inducing pyroptosis.^[^ ^123^ ^]^

GSDMA is located on chromosome 17 at the 17q21.1 region in humans, and on mouse chromosome 11D, there are three homologous genes encoding GSDMA1, GSDMA2, and GSDMA3.^[^ ^124^, ^125^ ^]^ It is mainly expressed in the skin, tongue, and stomach of the body and is associated with autoimmune diseases and cancer.^[^ ^126^ ^]^ The mouse GSDMA and GSDMA3 genes have functions in regulating epithelial maintenance and homeostasis, and human GSDMA and rat GSDMA, as direct homologs of mouse GSDMA, share commonalities and similarities.^[^ ^127^ ^]^ Knocking out GSDMA1 in mice weakens their immune response to bacteria, leading to uncontrolled bacterial infections and death. Additionally, variations, aging, and cardiovascular metabolic complications of GSDMA are one of the significant factors affecting the number and shape of neutrophils, but clonal hematopoietic stem cell proliferation does not affect it.^[^ ^128^ ^]^

GSDME is located at the 7p15.3 locus on chromosome 7 and is also known as deafness, autosomal dominant 5 (DFNA5).^[^ ^129^, ^130^ ^]^ It is important to note that the overexpression of GSDME is involved in the migration and invasion of tumors and is positively correlated with microvascular density.

PJVK (GSDMF) is located at 2q31.2 and consists of 352 amino acids. It is the first human gene associated with nonsyndromic hearing loss caused by neuronal defects.^[^ ^131^, ^132^ ^]^ Initially, PJVK was cloned from the human testis.^[^ ^94^ ^]^ Compared to other GSDM family members, PJVK exhibits differences in sequence homology, and structurally it is characterized by a short and non‐homologous C‐terminal domain, with no reports on its pore‐forming content to date.^[^ ^19^, ^129^, ^130^ ^]^ However, due to the short C‐terminal domain of PJVK, some researchers have considered whether PJVK lacks pyroptotic auto‐inhibitory activity and instead directly induces pore formation in peroxisomes to play a role in antioxidant stress. It has been proposed that PJVK is closely related to peroxisomes, which are key organelles in maintaining the redox homeostasis of the auditory system. Peroxisomes can regulate the excessive production of ROS induced by high‐level sound energy, and subsequently, PJVK can recruit autophagy‐related protein LC3B to peroxisomes in wild‐type mice, with autophagy mediating the early and rapid selective clearance of damaged peroxisomes in mouse auditory hair cells.^[^ ^94^, ^133^, ^134^ ^]^

#### 3.1.2 Gasdermins Pore Formation

The core event of pyroptosis is the pore formation of Gasdermins, which we will discuss later as various pre‐events leading to the formation of Gasdermin pores. As mentioned earlier, pore formation directly induces pyroptosis, and once these small pores are formed, there are early events of pyroptosis, such as ion flux and cell swelling, accompanied by mitochondrial depolarization and lysosomal leakage.^[^ ^135^ ^]^ Finally, before the cell ultimately lyses, a pyroptotic body with a diameter of 1–5 µm is formed.^[^ ^136^ ^]^

Taking GSDMD pore formation as an example, it has been resolved that the pores are larger than the already‐characterized GSDMA3 pores, consisting of 31 to 34 subunits. These GSDMD pores have a diameter of 1.1–2.4nm, and it is noteworthy that these pores directly induce pyroptosis, rather than relying on osmotic pressure to cause cell lysis.^[^ ^136^ ^]^ It can be inferred that, theoretically, the inner diameter of these pores is large enough to release IL‐1β and other DAMPs passively, and after the subsequent cell rupture, larger molecules such as lactate dehydrogenase can be released, and even organelles can be expelled.^[^ ^137^ ^]^ Therefore, the release of IL‐1β is dependent on the pore formation of GSDMD.^[^ ^138^ ^]^ It is important to note that the size of pore formation is concentration‐dependent on the expression of GSDMD‐NT, with higher expression of GSDMD‐NT leading to the formation of larger pre‐pore assemblies. Thus, some have suggested that by fine‐tuning the expression of GSDMD‐NT, one can precisely control the size of the pores and what types of cellular contents can be released.^[^ ^17^, ^18^ ^]^ Additionally, the Ragulator‐Rag complex is necessary for GSDMD pore formation and macrophage pyroptosis, primarily by promoting the oligomerization of GSDMD on the plasma membrane rather than facilitating membrane localization.^[^ ^139^, ^140^ ^]^ However, the specific mechanism of GSDMD pore formation remains to be further investigated.

In recent years, Gasdermins have become widely recognized as the primary executors of pyroptosis, but numerous studies have also indicated that the Gasdermin family is extensively involved in cancer‐related pathways, drug resistance, immune subtypes, tumor microenvironments, and cancer stemness, with close associations to cell differentiation, cell proliferation, cell death, mitochondrial homeostasis, and antimicrobial responses.^[^ ^141^, ^142^ ^]^ For instance, in neutrophils, there is a significant difference in the translocation of GSDMD post‐inflammatory activation compared to macrophages; N‐GSDMD is not transported to the plasma membrane to form pores, but instead, N‐GSDMD is transported to azurophilic granules and autophagosomes, releasing IL‐1β through an autophagy mechanism‐dependent pathway, which is unrelated to pyroptosis. Therefore, unlike macrophages, neutrophils have a significant ability to resist progression to GSDMD‐dependent pyroptotic lysis.^[^ ^117^ ^]^ Furthermore, it has been found that the absence of GSDMD in macrophages delays tissue recovery but has little impact on the local inflammatory microenvironment or the lytic cell pyroptosis process. Subsequent studies further determined that 11,12‐EET can enhance the FGF‐FGFR signaling pathway by regulating the liquid–liquid phase separation (LLPS) of fibroblast growth factor (FGF), thereby promoting the activation and proliferation of muscle stem cells (MuSC).^[^ ^143^ ^]^

### 3.2 Pre‐Gasdermin Pore Formation Event: Immune Activation Signals

#### 3.2.1 The Mediating Role of Inflammasomes and Caspases in Pyroptosis

**Figure 4:**
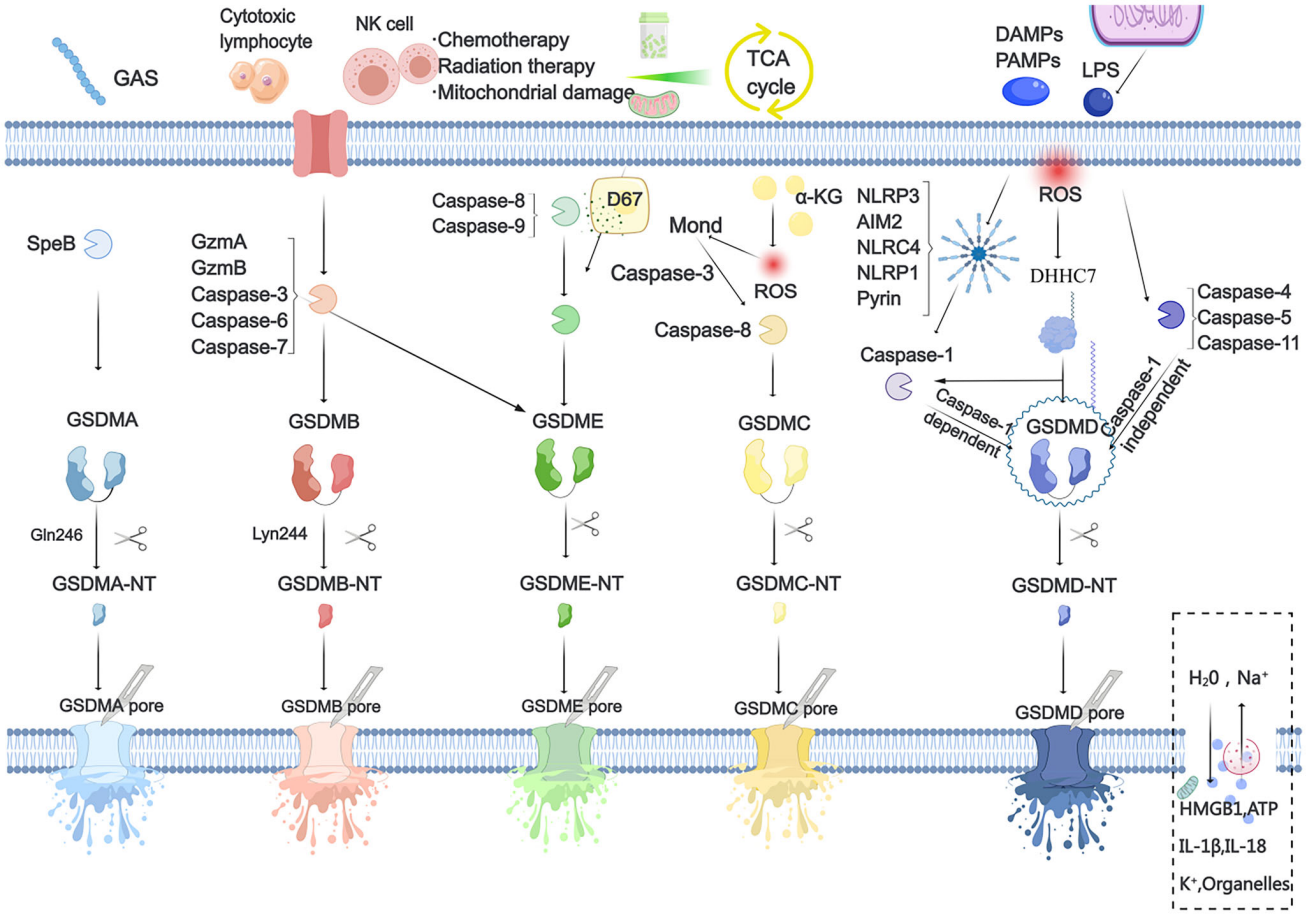
The molecular mechanism of pyroptosis, creating artboards in Medpeer.

**Figure 5:**
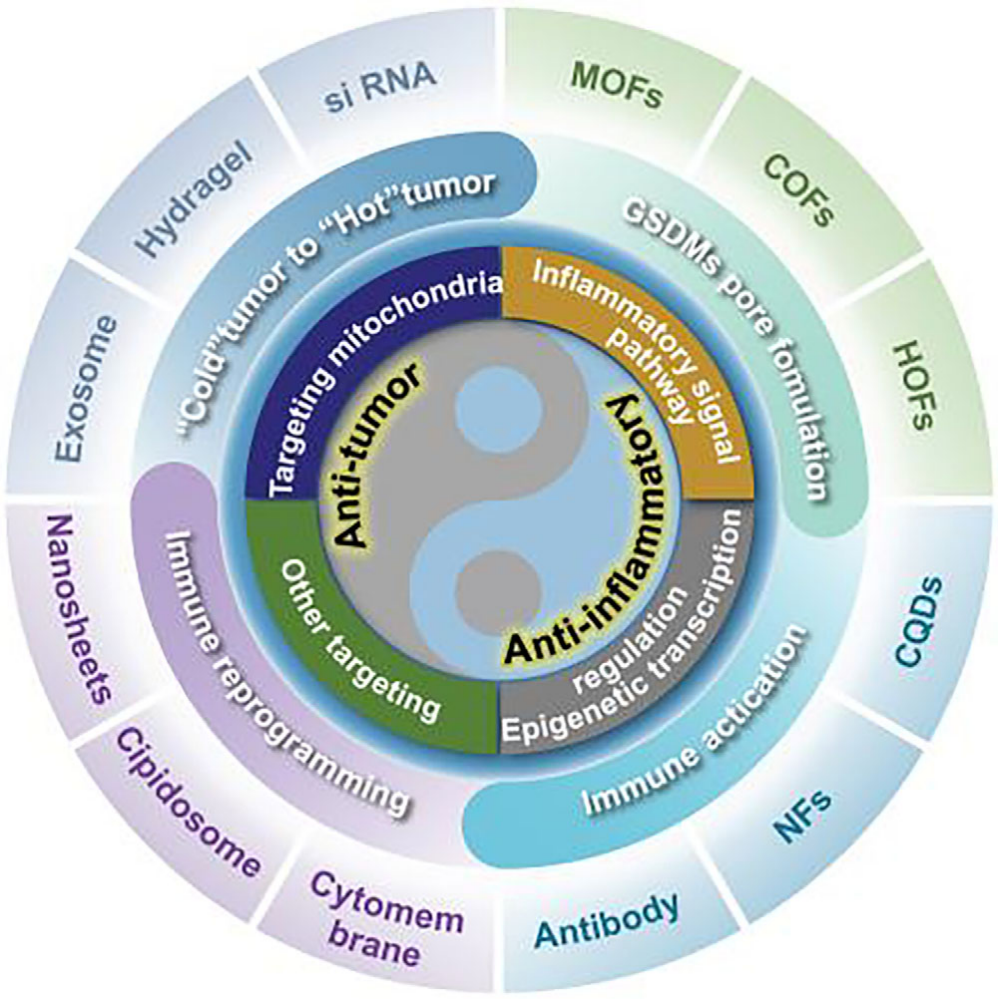
Scheme of biomaterials for targeting gasdermins, creating artboards in Adobe Illustrator.

As shown in **Figure** **4**, **5**, the pathway of pyroptosis begins with the inflammasome, which, upon sensing immune activation signals—pathogen‐associated molecular patterns (PAMPs), damage‐associated molecular patterns (DAMPs), and homeostasis‐altering molecular processes (HAMPs), activates Gasdermins.^[^ ^84^, ^144^, ^145^ ^]^ Pyroptosis induced by the GSDMs family can be divided into the canonical inflammasome pathway, the non‐canonical inflammasome pathway, and other pathways, depending on whether they depend on the inflammasome.^[^ ^146^ ^]^

The inflammasome is a multi‐protein complex that can be considered a signal transduction hub and serves as an activation platform for inflammatory caspases, typically composed of receptor proteins (NLR/ALR), adaptor proteins (ASC), and effector proteins pro‐Caspase, functionally responsible for activating inflammatory responses and playing an important role in the occurrence of innate immunity and inflammatory diseases.^[^ ^147^, ^148^, ^149^, ^150^ ^]^ Inflammatory Caspases, as the executioner family, mediate pyroptosis, including Caspases 1/4/5 in humans and 1/11 in mice.^[^ ^151^ ^]^

##### Classic Inflammasome Signaling Pathway

Most inflammasomes activate Caspase‐1, also known as the classic pathway.^[^ ^152^ ^]^ The classic inflammasome signaling pathway is a Caspase‐1‐dependent pyroptosis process triggered by the activation of the inflammasome. When the body detects the aforementioned immune activation signals, the inflammasome complex assembles, activating Caspase‐1, which in turn cleaves GSDMD, processes pro‐IL‐1β and pro‐IL‐18, and releases GSDMD‐NT to form pores on the cell membrane, leading to pyroptosis and the release of mature IL‐1β and IL‐18, as well as some cytokines.^[^ ^84^, ^144^, ^145^ ^]^

##### Non‐Canonical Inflammasome Signaling Pathway

The non‐classical inflammasome signaling pathway does not rely on inflammasome activation of Caspase‐1 but rather depends on pore formation by GSDMD.^[^ ^153^ ^]^ Lipopolysaccharides (LPS) from Gram‐negative bacteria can directly induce and trigger the activation of inflammasomes other than Caspase‐1, such as Caspase‐4, which undergoes intramolecular autoproteolysis at two sites: the C‐terminal of the p20 subunit and the N‐terminal of the p10 subunit. Caspase‐11 can also undergo specific autoprocessing at the N‐terminal of the p10 subunit. The p20/p10 heterodimer of Caspase‐4/11 can further form a tetramer, with intermolecular β‐sheet formations at the interface of the tetramer extending outward like a key, inserting into a hydrophobic pocket within the C‐terminal domain of GSDMD. This leads to the formation of a 2:2 enzyme‐substrate complex between Caspase‐4/11 and the C‐terminal domain of GSDMD. Therefore, activated Caspase needs to undergo precise autoprocessing at the N‐terminal of p10 to recognize and cleave GSDMD. As in the classical pathway, this ultimately leads to the formation of pores on the cell membrane and pyroptosis.^[^ ^81^, ^82^, ^154^ ^]^

It is worth noting that the differences between the non‐classical and classical inflammasome signaling pathways mainly lie in the activation mechanisms, Caspase isoforms, Gasdermin cleavage sites, and physiological functions. The classical pathway is primarily activated by pathogen‐associated molecular patterns (PAMPs) and damage‐associated molecular patterns (DAMPs) to activate Caspase‐1, while the non‐classical pathway is mainly triggered by LPS to activate inflammasomes other than Caspase‐1. Additionally, studies have shown that Caspase‐1 primarily cleaves at Asp276, while Caspase‐11 cleaves at both Asp285 and Asp276.^[^ ^155^ ^]^

##### Apoptotic Caspase Cleavage Activity

However, later studies also discovered the cleavage effects of some apoptotic caspases, such as the effector molecule YopJ produced by Yersinia, which can inhibit TAK1‐IκB kinase signaling while leading to Caspase‐8‐mediated GSDMD pore formation. TNF can also induce Caspase‐8‐dependent cleavage of GSDMD.^[^ ^156^, ^157^ ^]^ Moreover, during Yersinia infection, the Rag‐Ragulator can serve as a functional platform to recruit and activate RIPK1 and Caspase‐8, thereby triggering the cleavage and activation of GSDMD.^[^ ^139^, ^140^, ^158^ ^]^ Caspase‐3 has long been considered a key molecule in apoptosis; however, recent studies have found that it can be targeted and regulated by TNF or chemotherapeutic drugs, thereby competitively inhibiting its combination with apoptotic substrates and then cleaving the Asp270 site of GSDME, converting the relatively slow non‐inflammatory apoptosis into a faster inflammatory pyroptosis. It is worth noting that pyroptosis is also one of the important reasons for the side effects of some chemotherapeutic drugs.^[^ ^159^ ^]^ PD‐L1 usually binds to its receptor on the cell membrane as an immune checkpoint to play a role in tumor immunity; however, it is important to note another non‐immune checkpoint function of PD‐L1, which is mediated by GSDMC to convert TNF‐α‐induced apoptosis in breast cancer cells to pyroptosis, while promoting tumor necrosis.^[^ ^160^, ^161^ ^]^ In addition, mutated Caspase‐8 (C362A) induces the formation of ASC speck‐like proteins, and in the intestines of MLKL‐deficient mice around embryonic day 18, it leads to Caspase‐1‐dependent cleavage of GSDMD, Caspase 3, and Caspase 7.^[^ ^162^ ^]^

Furthermore, the triggering mechanism of cytokine release syndrome, a complication of CAR‐T therapy for cancer, has also been explained in the study of pyroptosis. In tumor cells, CAR T cells can activate Caspase‐3 by releasing granzyme B, leading to the cleavage of GSDME and thus causing cytokine release syndrome.^[^ ^163^ ^]^ Additionally, Li et al. found in sepsis models induced by *E. coli* and *Salmonella typhimurium* infection that inhibiting the Caspase‐7/GSDMB axis can lead to an increase in pyroptosis.^[^ ^164^ ^]^ Moreover, the metabolic product of the tricarboxylic acid cycle, α‐KG, is reduced to another metabolite, L‐2HG, in an acidic environment, which increases ROS expression and induces the oxidation and internalization of the plasma membrane‐localized death receptor DR6. Internalized DR6 recruits the Caspase‐8 precursor and GSDMC to the DR6 receptor, and the activated Caspase‐8‐mediated cleavage of GSDMC can induce pyroptosis.^[^ ^165^ ^]^

Furthermore, it is worth noting that different Caspases cleave different sites on Gasdermins, which may block pyroptosis. Caspase‐3/7, in apoptosis, specifically blocks pyroptosis by cleaving GSDMD at sites different from inflammatory Caspases.^[^ ^166^ ^]^

##### Other Signaling Pathways

In addition to the two traditional pathways, several other pyroptosis signaling pathways have been discovered. For example, the cysteine protease virulence factor SpeB of Streptococcus pyogenes (GAS) can autocatalytically cleave to produce an active protease to target the cleavage of GSDMA at the Gln246 site, leading to the release of the N‐terminal fragment of GSDMA, which can insert into the membrane to form lytic pores.^[^ ^87^, ^167^, ^168^ ^]^ However, there are dissenting opinions that argue that the pyroptosis mechanism of GSDMA is different from that of GSDMD, suggesting that GSDMA pyroptosis is preferentially localized to the mitochondrial membrane rather than the cytoplasmic membrane, with its accumulation on the plasma membrane being delayed and reduced, thus GSDMA‐N‐term leads to early mitochondrial dysfunction associated with plasma membrane permeabilization and regulated necrosis.^[^ ^169^ ^]^ Additionally, granzyme B has also been found to directly cleave and activate GSDME at the D270 site.^[^ ^170^ ^]^ Another member of the granzyme family, granzyme A from Cytotoxic T cells, has also been shown to cleave GSDMB at the Lys244 site to release the N‐terminal domain.^[^ ^86^ ^]^

#### 3.2.2 Mitochondrial Homeostasis: Reprogramming of Cell Death

Recent studies have found that mitochondrial dysfunction plays a crucial role in the induction and regulation of pyroptosis, and altering mitochondrial homeostasis can reprogram cell death patterns. Studies have shown that the Gasdermin family can also form pores within mitochondria. When the inflammasome is activated, mitochondrial ROS can guide GSDMD to the mitochondria, where it binds to the mitochondrial membrane. After the formation of mitochondrial GSDMD pores and the release of mtROS, mtROS can promote the shift from pyroptosis to RIPK1/RIPK3/MLKL‐dependent necroptosis, causing excessive inflammation and severe immunopathology.^[^ ^171^ ^]^Additionally, released mtDNA can promote GSDMD pore formation, and studies have shown that AIM2 inflammasomes can also sense mtDNA and activate pyroptosis pathways.^[^ ^135^, ^172^ ^]^ Furthermore, GSDMD can regulate the AMPK/PGC‐1α pathway to target Aifm3, participating in the organization of the mitochondrial electron transport chain. This process promotes the shift from oxidative phosphorylation (OXPHOS) to glycolysis.^[^ ^173^ ^]^ It is evident that regulating mitochondrial homeostasis plays a significant role in controlling pyroptosis, and many nanomedicines are currently designed based on mitochondrial dysfunction.

#### 3.2.3 Transcriptional and Epigenetic Functions of Gasdermins

As mentioned earlier, the method by which Gasdermins execute pyroptosis is through pore formation, and thus, the expression levels of Gasdermins are highly correlated with the extent of pyroptosis. However, not all Gasdermins lead to cell death after being cleaved. A significant portion of cells with active inflammasomes are resistant to pyroptosis. In other words, when GSDMD cleavage induces pyroptotic pores, calcium ion influx recruits the required endosomal sorting complexes to the damaged membrane areas as a signal for the cell to initiate membrane repair.^[^ ^174^ ^]^ Consequently, the expression levels of Gasdermins are crucial in determining whether the degree of pore formation can exceed the extent of membrane repair, thereby triggering pyroptosis. Modulating the expression of Gasdermins has become an effective strategy for regulating cell death and cytokine release.^[^ ^19^ ^]^

##### Palmitoylation

Palmitoylation is a post‐translational modification involving the covalent attachment of long‐chain fatty acids (typically palmitic acid with 16 carbons) to protein cysteine residues through thioester bonds. This modification is a dynamic and reversible process, often involving two stages of autoacylation and transfer. Recent studies have shown that human GSDMD‐N‐terminal Cys191 (mouse Cys192) can promote pyroptosis through reversible S‐palmitoylation. Notably, cleavage is no longer the only trigger for GSDMD, and palmitoylation is required for pore formation. ZDHHC5, ZDHHC9, and DHHC7 are the main palmitoylating enzymes for GSDMD, while APT2 regulates depalmitoylation. ROS produced by mitochondria can promote S‐palmitoylation. Palmitoylation can enhance the cleavage function of GSDMD and promote the generation of GSDMD‐NT and its oligomerization and membrane translocation. Additionally, palmitoylation can enhance the binding of GSDMD‐NT to phosphatidylinositol and cardiolipin, regulate the localization of GSDMD‐NT on the plasma membrane, and affect the oligomerization of GSDMD‐NT. Moreover, reversible palmitoylation has been reported as a checkpoint for the formation of pores by GSDMD‐NT and full‐length GSDMD and serves as a universal switch for the activation of the GSDMD family.^[^ ^47^, ^175^, ^176^, ^177^, ^178^ ^]^

##### Methylation

Methylation is an epigenetic modification involving the addition of a methyl group to specific chemical groups on DNA, RNA, or proteins. This modification can regulate gene expression without altering the DNA sequence. Studies on Gasdermins regulated by methylation are numerous. Changes in Gasdermins can lead to alterations in the physiological and pathological conditions at the gene's location. The human 17q12‐21 gene locus is associated with GSDMA and asthma, leading to changes in the expression levels of the GSDMA gene in CD4 T cells of asthma patients and affecting its methylation levels. Suitable GSDMA promoter methylation changes can alter the expression of GSDMA.^[^ ^128^, ^179^, ^180^ ^]^ Furthermore, the silencing of N6‐adenosine methyltransferase Mettl14 disrupts the m6A modification of GSDMC, leading to reduced expression of GSDMC, impaired mitochondrial function, apoptosis of Lgr5+ stem cells, and difficulty in maintaining normal colonic epithelial morphology.^[^ ^181^ ^]^ Notably, due to the expression of GSDME in tumor sites, methylation regulation of GSDME is more common in various nanomedicine studies.

##### Transcription

Recent studies have shown that IRF2 in mouse macrophages induces the expression of GSDMD at the transcriptional level, with the factor selectively binding to the transcription start site of GSDMD to reduce its expression.^[^ ^182^ ^]^ It is important to note that human monocytes do not rely on IRF2 for the regulation of GSDMD expression.^[^ ^183^ ^]^ Currently, there is still a lack of research on the transcriptional mechanisms of the Gasdermins family, which warrants further investigation.

#### 3.2.4 Approved Pyroptosis Regulator

The inhibition or activation of GSDMD can be applied to different disease treatment strategies, such as cancer immunotherapy that increases GSDMD expression and activates it, and anti‐inflammatory therapy that suppresses GSDMD expression to inhibit inflammatory cell death. Therefore, drugs targeting the regulation of Gasdermins are a promising therapeutic strategy, and we outline several different GSDMD inhibitors and activators here.

##### GSDMD Inhibitor

Disulfiram can act as a pyroptosis inhibitor, covalently modifying human GSDMD at Cys191/mouse GSDMD at Cys192 to prevent the formation of GSDMD pores, thereby preventing cell pyroptosis and IL‐1β release, although the processing of IL‐1β and GSDMD can still occur.^[^ ^91^ ^]^ Similarly, dimethyl fumarate delivered to cells or endogenous fumarate can modify Cys192 of GSDMD, reacting with GSDMD at the key cysteine residue to form S‐(2‐succinyl)‐cysteine, whose succinylation organizes its interaction with caspases, limiting its ability to induce pyroptosis and can be used as a pyroptosis inhibitor.^[^ ^92^ ^]^ These small molecules can react with the free thiol group at the (Cys191/192) site of GSDMD, thereby blocking pore formation and pyroptosis.^[^ ^11^ ^]^ The two small molecule inhibitors have been approved by the FDA. The Shigella flexneri ubiquitin ligase IpaH7.8 can act as a specific inhibitor for human GSDMD, promoting species‐specific degradation by ubiquitinating GSDMD and targeting it for proteasomal degradation.^[^ ^184^ ^]^ NU6300 is also a specific GSDMD inhibitor that acts on human GSDMD‐C191, impairing the palmitoylation of full‐length and N‐terminal GSDMD, blocking the membrane localization and oligomerization of N‐terminal GSDMD, and exerting GSDMD inhibitory effects. It does not affect the early steps of AIM2 and NLRC4‐mediated inflammation, such as ASC oligomerization and caspase‐1 processing. In contrast, NU6300 strongly inhibits these early steps in NLRP3 inflammasomes.^[^ ^16^ ^]^ Additionally, the protein tyrosine phosphatase PtpB from Mycobacterium tuberculosis can dephosphorylate phosphatidylinositol‐4‐monophosphate and phosphatidylinositol‐(4,5)‐bisphosphate on the host cell membrane, thereby disrupting the membrane localization of GSDMD and inhibiting macrophage pyroptosis.^[^ ^185^ ^]^In addition to disulfiram, dimethyl fumarate, and necrosulfonamide—small‐molecule inhibitors targeting oligomerization interface III—recent studies have identified two novel GSDMD inhibitors, SCR‐1481B1 and cefcapene pivoxil hydrochloride hydrate (repurposed compounds), which specifically inhibit GSDMD‐induced pyroptosis by targeting the essential N‐terminal oligomerization interface I. Furthermore, research demonstrates a synergistic (1+1>2) dual‐target therapeutic effect between these inhibitors, effectively suppressing GSDMD‐mediated pyroptosis and tumor progression in septic mouse models.^[^ ^186^ ^]^

##### GSDMD Agonist

The tripartite motif protein TRIM21 can act as a positive regulator of GSDMD‐dependent pyroptosis. It interacts with GSDMD through the PRY‐SPRY domain, maintaining the stable expression of GSDMD in resting cells and inducing the aggregation of GSDMD‐NT during pyroptosis.^[^ ^187^ ^]^ DMB is a direct and selective small molecule GSDMD agonist that can bind to the Cys191 site of GSDMD without cleaving GSDMD, then forming GSDMD pores, and can promote low‐level pyroptosis in combination with PD‐L1 therapy in multiple tumor models without toxicity.^[^ ^9^ ^]^ In experiments with multidrug‐resistant Acinetobacter baumannii, type I interferon induced the expression of genes Zbp1, Mlkl, caspase‐11, and GSDMD through H3K27ac modification, activating NLRP3 inflammasomes and potentially leading to GSDMD‐mediated pyroptosis.^[^ ^188^ ^]^

However, as previously mentioned, the Gasdermin family is not limited to pyroptotic functions and also possesses non‐pyroptotic functions. The upregulation of some genes can also accelerate the occurrence and development of tumors. For instance, the overexpression of GSDME is involved in tumor migration and invasion and is positively correlated with microvascular density.^[^ ^129^ ^]^ Additionally, in the cortical development of adult mice, pyroptosis mediated by GSDMD occurs in response to high levels of replicative stress, aiding in the elimination of damaged neural progenitor cells. A deficiency can lead to defects in the DNA damage sensor pathway, accompanied by neurogenesis and autism‐like behaviors.^[^ ^189^ ^]^ Therefore, when designing drugs that directly regulate Gasdermins, this issue should be taken into account. Furthermore, diseases involve various types of cell death, and simply regulating the expression of GSDMD to control the process of pyroptosis may not be effective in many diseases. It is possible to consider the combined application with other cell death pathways in the design.

### 3.3 Post‐Gasdermin Pore Formation Event: Immune Reprogramming

The core characteristic of pyroptosis is its inflammatory mode of cell death. After the formation of Gasdermin pores, cellular contents marked by IL‐1β and IL‐18 will be released, and the processing of IL‐1β can, in turn, amplify the aforementioned pathways to accelerate inflammatory cascades, leading to local inflammation, activation of the immune response, and currently being widely applied in tumor immunotherapy, anti‐inflammatory therapy, and antibacterial therapy.

#### 3.3.1 Immune Reprogramming in the Tumor Microenvironment

Emerging tumor immunotherapies have replaced traditional tumor treatment methods and have become the mainstream cancer therapy. Based on the spatial distribution of immune cells in the tumor microenvironment, tumors can be divided into three basic immune phenotypes: immune‐inflammatory, immune‐excluded, and immune‐desert types.^[^ ^190^ ^]^ The immune‐excluded and immune‐desert types are considered “cold tumors,” characterized by poor T‐cell infiltration, suppression of T‐cell activation, and the presence of immune‐suppressive cell populations.^[^ ^191^, ^192^, ^193^ ^]^ Since immune‐inflammatory tumors, or “hot tumors,” have more active immune cells, the application of cancer immunotherapy is effective.

Conversely, immune cells find it difficult to act on “cold tumors,” posing challenges for cancer immunotherapy. However, after pore formation due to pyroptosis, the release of inflammatory cytokines such as IL‐1β and IL‐18 induces local inflammation, triggers the immune response, and amplifies the immune reaction, converting “cold tumors” into “hot tumors.” Therefore, the activation of pyroptosis pathways can target the tumor microenvironment for immune reprogramming to treat cancer. Additionally, monotherapy is prone to drug resistance in tumor treatment, and the mutation spectrum of solid tumors is dynamically changing, with new mutations continuously emerging and some old mutations disappearing, making monotherapy inevitably limited. Therefore, the design of tumor immunotherapy can consider the synergistic effect of multiple therapies. However, it should be noted that long‐term inflammation may lead to the occurrence and development of tumors.

#### 3.3.2 Immune Reprogramming in the Inflammatory Microenvironment

From the 19th century to the present, scientists have continuously proposed the association between tumors and inflammation, including theories that tumors may originate from chronic inflammation and that the inflammatory wound healing process may promote the production of tumor stroma. Modern pathological research has proven that intra‐tumoral leukocyte infiltration has become a common marker of tumors, also proving the innumerable connections between tumors and inflammation.^[^ ^194^, ^195^, ^196^, ^197^ ^]^

Inflammation, as an important pathological process, is a protective response of the body to external stimuli. Appropriate inflammation can maintain homeostasis, but persistent chronic inflammation can lead to many inflammatory diseases and even the occurrence and development of tumors. For example, gastric cancer is related to Helicobacter pylori infection, nasopharyngeal cancer is related to herpes virus infection, liver cancer is related to hepatitis virus infection; obesity and depression, and other systemic inflammations are also related to the incidence of tumors and poor efficacy of anti‐cancer treatment.^[^ ^198^ ^]^ Conversely, timely control of inflammation is conducive to the treatment and recovery of diseases. As mentioned earlier, pyroptosis is an inflammatory mode of cell death, mainly releasing pro‐inflammatory cytokines through pore formation, playing a crucial role when the host encounters external stimuli. It is worth noting that pyroptosis is a “double‐edged sword,” and excessive inflammation caused by pyroptosis may exacerbate the development of inflammatory diseases and over‐activation of the immune system and may even lead to “cytokine storms,” harmful inflammation, or even organ dysfunction.^[^ ^13^, ^14^, ^15^, ^16^ ^]^ Therefore, the design strategy for anti‐inflammatory drugs involving pyroptosis is mostly inhibitors of the pyroptosis pathway, starting from the perspective of suppressing harmful inflammation, and the strategy of expanding the immune response by inflammation is mostly applied in cancer treatment.

#### 3.3.3 Immune Reprogramming in Antimicrobial Defense

Although Gasdermins were initially discovered in mammals, recent studies have shown that GSDMD can also mediate the death of bacteria and fungi.^[^ ^18^, ^178^ ^]^ Active GSDMD‐NT kills bacteria by directly interacting with cardiolipin, and the Glu236 residue of GSDMD is the truly important site for bacterial recognition of GSDMD.^[^ ^199^ ^]^ Wild‐type GSDMD‐NT can quickly bind to both Gram‐positive and Gram‐negative bacteria and kill them, but other GSDMDs that do not expose the N‐terminal residues, such as full‐length and GSDMD‐CT, do not have this ability.^[^ ^118^, ^119^ ^]^ Additionally, studies have shown that GSDMD exerts its antibacterial effect by preventing Candida albicans from escaping from macrophages and can maintain inflammasome dependence without relying on IL‐1β production to combat fungal host defense, thereby exerting its antibacterial ability.^[^ ^200^ ^]^ It should be noted that after pyroptosis, live bacteria in macrophages are not released but are captured by cellular traps (pits) and promote phagocytic signaling to drive inflammasome detection and supply neutrophils to clear bacteria within cells.^[^ ^201^ ^]^ The above studies have proven the antibacterial ability of GSDMD, but its antibacterial effect on different bacteria and the specific mechanisms of action still require further research.

## 4 Biomaterials for Targeting Gasdermins

Gasdermins have been confirmed to play a unique role in the drug development for tumor immunotherapy and immune‐related inflammatory diseases. However, as mentioned above, pyroptosis plays a significant role in regulating inflammation, but it is easy to cause overvalidation, so excessive inflammation should be considered in drug design. Moreover, long‐term single‐drug strategies are prone to the development of drug resistance. Therefore, intelligent composite nanomaterials can link the uniqueness of a variety of materials and collaborate to improve the therapeutic effect against drug resistance, and they have the advantages of shape control, spatio–temporal selectivity, high targeting, low immunogenicity, and prevention of cytokine storms. To enhance their targeting of tumors or inflammatory tissues, nanodrugs are generally designed with stimulus‐responsive strategies based on the physiological characteristics of the tumor microenvironment or inflammatory microenvironment, such as the hypoxic state, weakly acidic environment, and redox homeostasis imbalance caused by overexpression of glutathione in the tumor microenvironment, as well as the high expression of certain specific enzymes; and excessive ROS expression in inflammatory tissues. The potential of functional materials in targeted positioning and precise regulation of Gasdermins' functions is enormous, indicating a broad prospect for their clinical translation.

### 4.1 Targeted Regulation of Gasdermin Pores

**Figure 6:**
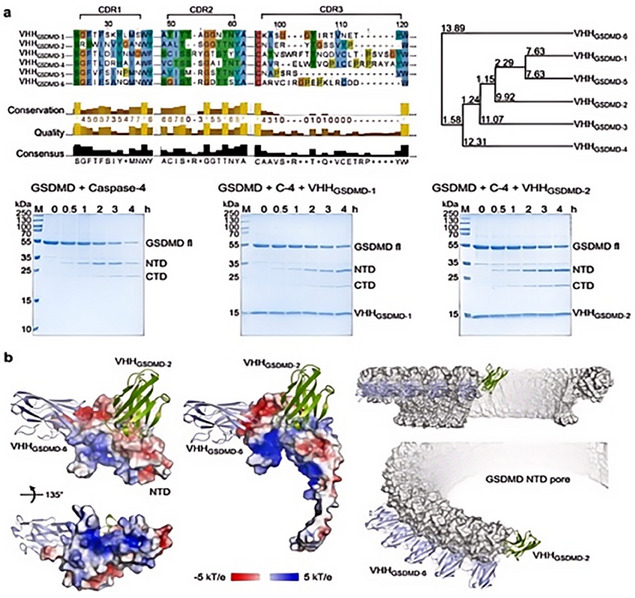
a) Sequence alignment of six GSDMD‐specific nanobodies. b) Visualization of binding modes between six GSDMD‐specific nanobodies and GSDMD. Reproduced with permission.^[^ ^203^ ^]^ Copyright 2024, Springer Nature.

The key to whether the pyroptosis pathway can be activated lies in the pivotal event – pore formation, which is designed to have pore‐forming inhibitory drugs for the treatment of inflammatory diseases. As mentioned earlier, there are three small molecule GSDMD inhibitors that have been marketed: necrosulfonamide, disulfiram, and dimethyl fumarate, all of which inhibit pore formation by covalently modifying the C191 site of GSDMD‐NT. However, they share a common problem in that they do not specifically bind to GSDMD, which may bring harmful off‐target effects to the body. To address the aforementioned issue, researchers used full‐length recombinant human GSDMD protein as an antigen to stimulate the immune system of llamas and selected six nanobodies, as shown in **Figure** **6**, which were found to inhibit the oligomerization of GSDMD‐NT to block the formation of GSDMD pores, rather than inhibiting the cleavage to form them. The development of these specifically targeted nanobodies provides support for the design and application strategies of biomaterials later on.^[^ ^18^, ^202^, ^203^ ^]^

Furthermore, as we mentioned earlier, the method of membrane repair is that calcium ions enter the cell interior through the pores to recruit ESCRT‐III to repair the cell membrane. Therefore, increasing the concentration of calcium ions in the local inflammatory area during pyroptosis may provide anti‐inflammatory effects for inflammatory diseases.

The above examples show that the potential of functional materials designed for Gasdermins‐mediated pore formation, the core event of pyroptosis in cancer and inflammatory diseases, deserves attention. Gasdermin‐mediated pores can be visually regarded as the switch of inflammation. At present, the research on turning on this switch to transform immune desert tumor into immune infiltrating tumor is extensive, but the functional materials that turn off this switch to block inflammatory pathways and thus treat inflammatory diseases still need further research.

### 4.2 Targeted Regulation of Immune Activation Signals Prior to Pore Formation

#### 4.2.1 Classic, Non‐Canonical Inflammasome Signaling Pathways and Other Signaling Pathways

##### Classic, Non‐Canonical Inflammasome Signaling Pathways

Most natural materials regulate the inflammatory microenvironment by affecting GSDMD and Caspase‐1/11 and their upstream and downstream proteins to exert anti‐inflammatory or pro‐inflammatory effects.^[^ ^143^, ^144^, ^145^ ^]^

**Figure 7:**
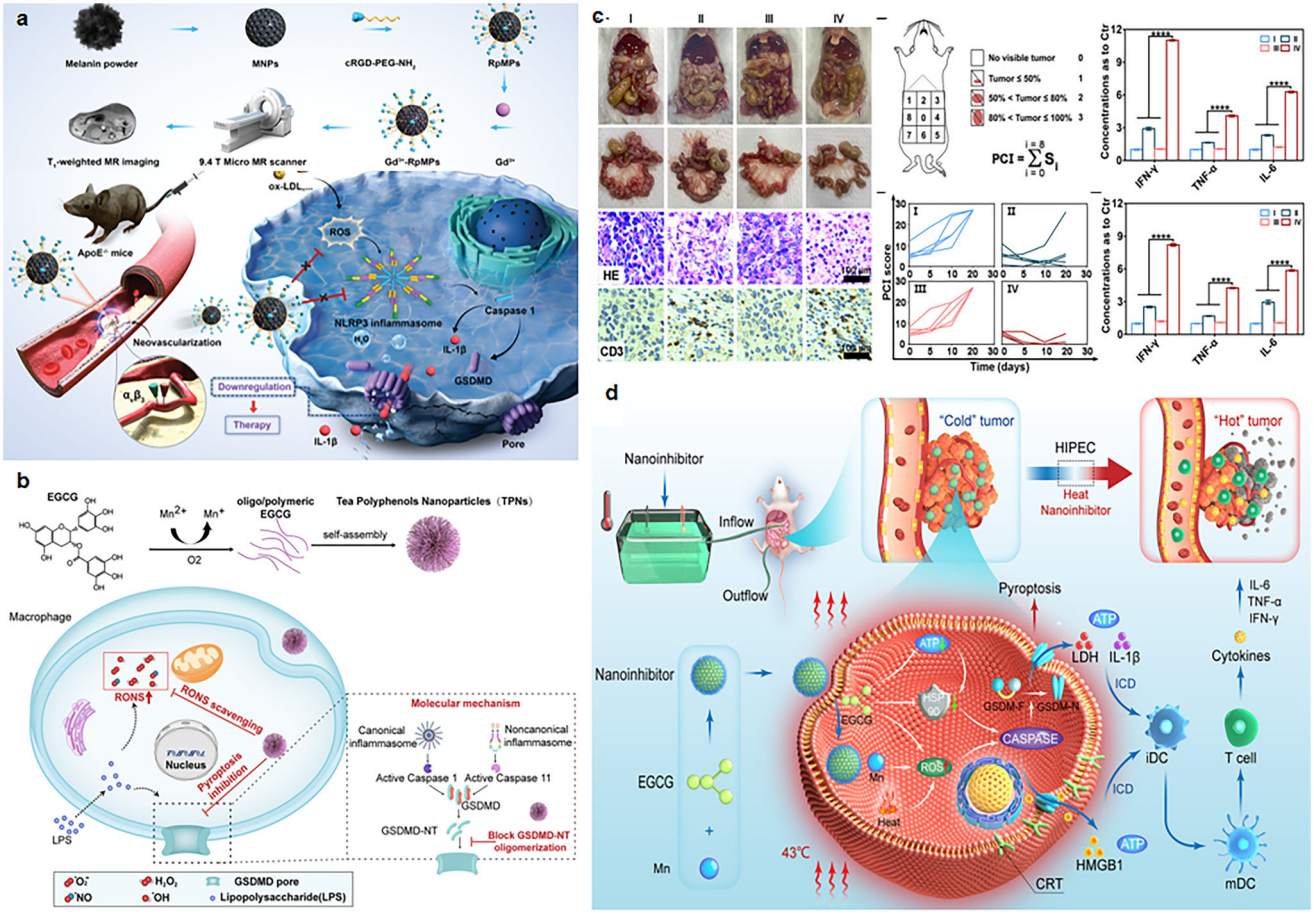
The working content of pyroptosis regulators in canonical and Non‐canonical inflammasome signaling pathways. a) Strategy of ultrasmall melanin nanoparticles. Reproduced with permission.^[^ ^42^ ^]^ Copyright 2023, Wiley. b) Schematic diagram of drug‐free catechin nanoparticles strategy. Reproduced with permission.^[^ ^43^ ^]^ Copyright 2022, ACS. c) Pharmacodynamic study of carrier‐free self‐assembled nanodrugs. d) Strategy diagram of carrier‐free self‐assembled nanodrugs. Reproduced with permission.^[^ ^78^ ^]^ Copyright 2023, Cell Press.

At present, the design strategy of nanomedicine targeting the regulation of immune activation signals before pore formation has tended to be multi‐pathway synergistic. For instance, as shown in **Figure** **7**, Zu et al. designed an ultrasmall melanin nanoparticle resembling a cellular patch that targets the formation of neovessels in plaques. By clearing ROS within LPS‐induced macrophages, it inhibits pyroptosis and alleviates inflammatory responses, aiding in the treatment of atherosclerosis through the classical inflammasome signaling pathway NLRP3‐Caspase 1‐GSDMD.^[^ ^42^ ^]^ Sepsis is a life‐threatening disease caused by the dysregulation of the host's immune response to infection, characterized by the overproduction of various reactive oxygen and nitrogen species (RONS) and excessive pyroptosis. To address pyroptosis induced by sepsis, Chen et al. designed a drug‐free catechin nanoparticle with RONS clearance activity. Additionally, it possesses the ability to inhibit GSDMD pore formation to combat pyroptosis, exerting its effects through both the classical inflammasome signaling pathway Caspase‐1 and the non‐canonical inflammasome signaling pathway Caspase‐11. This nano‐drug design employs a multi‐faceted strategy against sepsis, providing a reference for the design of drugs targeting diseases with immune dysregulation.^[^ ^43^ ^]^ The high expression of heat shock protein can produce heat resistance in the treatment of clinical abdominal thermoperfusion chemotherapy. Therefore, Wang et al designed a carrier free self‐assembled nanomedical drug composed of Mn ion and EGCG, which can directly inhibit HSP90 and damage the HSP90 chaperone cycle by reducing the level of intracellular ATP. At the same time, heat and Mn ions can synergistically lead to oxidative stress and the classical inflammasome signaling pathway Caspase‐1‐GSDMD.^[^ ^78^ ^]^ This “two birds with one stone” strategy can provide ideas and references for cancer immunotherapy to collaborate with other approaches.

##### Other Signaling Pathways

Some natural materials exert their therapeutic effects by regulating GSDME and Caspase‐3/9 and their upstream and downstream proteins.^[^ ^25^, ^29^, ^35^ ^]^

**Figure 8:**
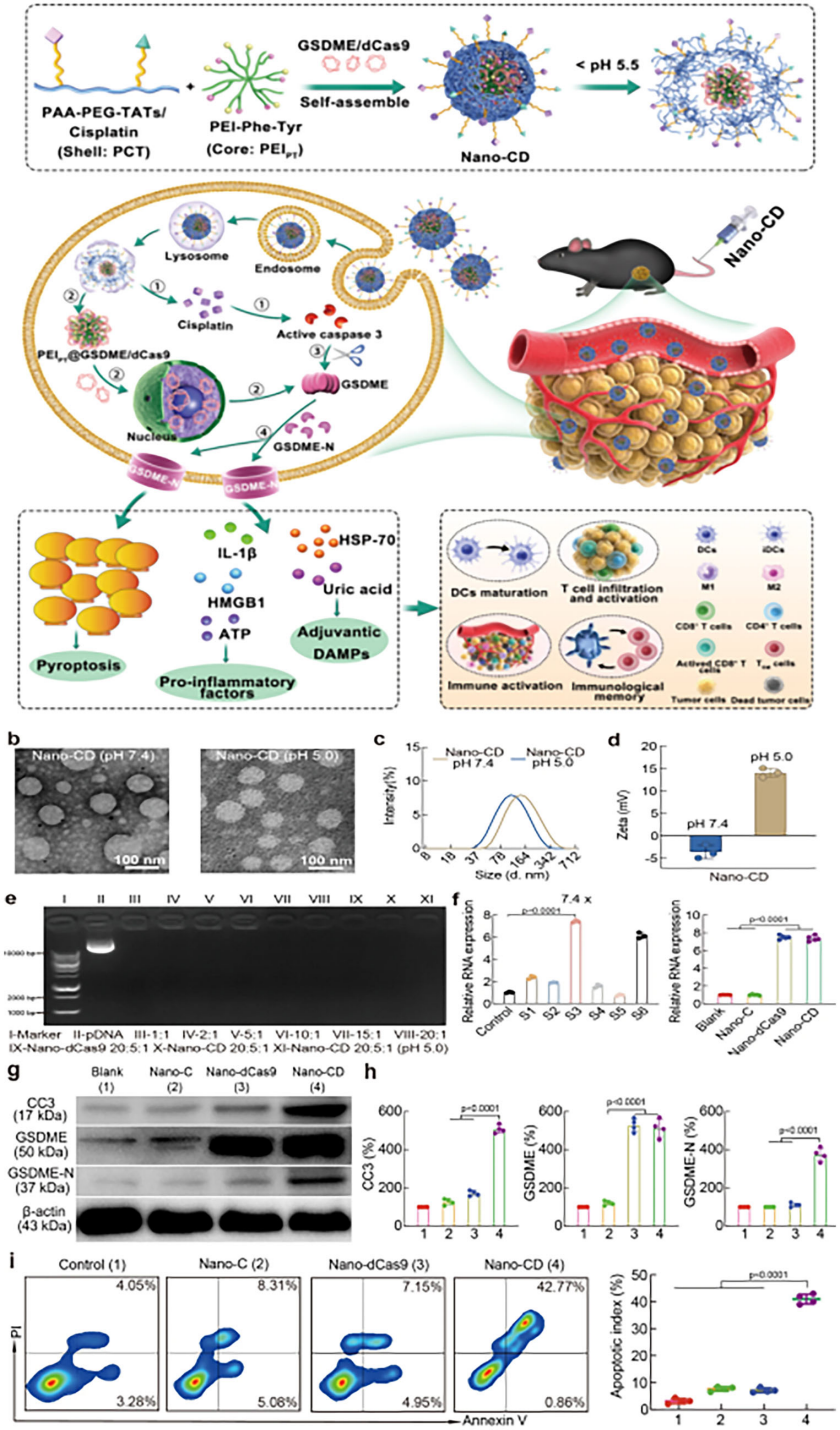
The working content of pyroptosis regulators in other signaling pathways. Reproduced with permission.^[^ ^56^ ^]^ Copyright 2023, Springer Nature.

The immune response induced by solely activating the pyroptosis of the Gasdermins family within the body is sometimes insufficient for antitumor effects. In light of the aforementioned situation, as shown in **Figure** **8**, Wang and colleagues designed a Nano‐CRISPR scaffold that utilizes a specific sgRNA selected from functional screening to trigger the expression of endogenous GSDME, release cisplatin to initiate immune cell death, and to prevent capture by lysosomes, polyethylene imine was encapsulated outside the chemotherapeutic drugs and plasmids, successfully escaping from endosomes and successfully releasing cisplatin and plasmids in the acidic tumor microenvironment. Mechanistically, it is also designed from the apoptotic Caspase‐3‐GSDME signal pathway. This novel pyroptosis inducer and the self‐supplied GSDME combination reverse the immunosuppressive tumor microenvironment and amplify the adaptive antitumor immune cascade. This method of achieving protein self‐sufficiency in tumor cells provides a new strategy for the development of tumor drugs.^[^ ^56^ ^]^

#### 4.2.2 Transcription and Epigenetic Regulation

In recent years, extensive research has clarified the significant anti‐inflammatory, antitumor, and immunomodulatory effects of nanomaterials that target the transcription and epigenetics of Gasdermins. The specific biological activity design of nanomaterials is intricately related to their targeting sites. To simplify the development of targeted regulation of Gasdermins transcription and epigenetics, we categorize their targeting strategies here into targeting methylation, palmitoylation, etc.

Natural materials mainly consist of natural products with methyltransferase inhibitory activity, such as EGCG.^[^ ^51^ ^]^

**Figure 9:**
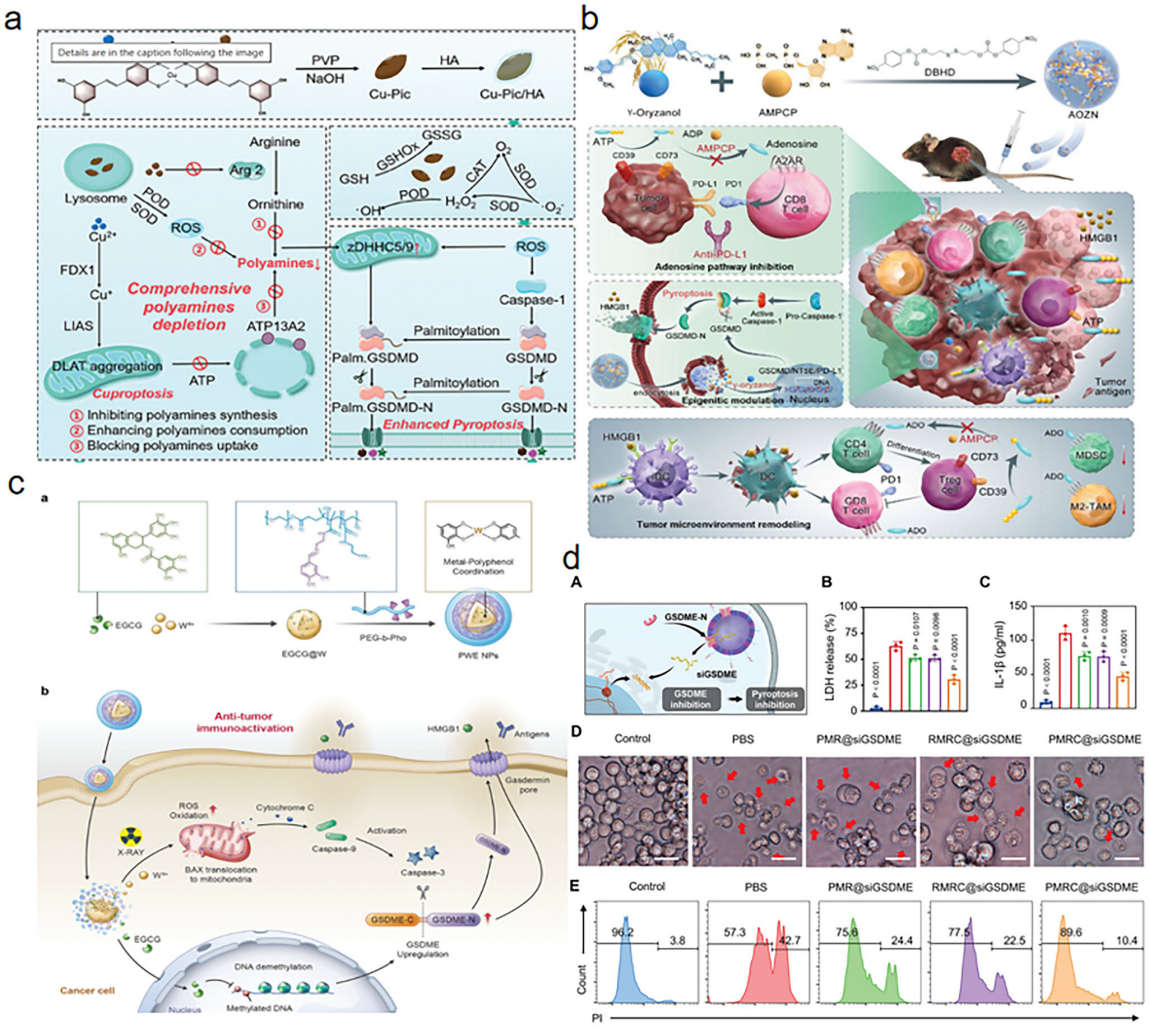
The working content of epigenetic pyroptosis regulators. a) Multifunctional Cu‐Pic/HA NPs mechanism schematic. Reproduced with permission.^[^ ^61^ ^]^ Copyright 2024, Wiley. b) Prodrug nanomicelles mechanism schematic. Reproduced with permission.^[^ ^62^ ^]^ Copyright 20121, Wiley. c) Pyroptosis‐promoting nanodrug mechanism schematic via combined strategy of DNA methyltransferase inhibitors and X‐ray‐responsive ROS amplifiers. Reproduced with permission.^[^ ^51^ ^]^ Copyright 2023, Wiley. d) Spatiotemporally selective siRNA delivery system in vitro pharmacodynamic profile. Reproduced with permission.^[^ ^79^ ^]^ Copyright 2024, Wiley.

Currently, the main epigenetic regulation strategy targeting pyroptosis is through the inhibition of DNA methylation to exert antitumor immune effects. As shown in **Figure** **9**, Zhu et al. designed a multifunctional copper‐paclitaxel/hyaluronic acid nanoparticle (Cu‐Pic/HA NPs) based on the epigenetic regulation of gasdermins in the tumor immune microenvironment. The drug targets the overall depletion of polyamines within cells, leading to mitochondrial dysfunction and an increase in copper ions, thereby causing an increase in ROS expression within mitochondria, further upregulating the expression of zDHHC5 and zDHHC9, promoting the palmitoylation of GSDMD and GSDMD‐N, thus inducing pyroptosis and copper death reactions to achieve antitumor treatment.^[^ ^61^ ^]^

Due to the methylation of the GSDME gene promoter, the expression level of GSDME in tumor cells is much lower than in normal cells. Therefore, among the Gasdermin family, the target protein for nanomaterials to regulate the level of pyroptosis through epigenetic strategies is mainly GSDME.^[^ ^204^ ^]^ For example, Xiong et al. designed a prodrug nano‐micelle with the main components of the epigenetic regulator γ‐oryzanol (Orz), adenosine inhibitor α,β‐methylene adenosine 5′‐diphosphate (AMPCP) to address the characteristics of immune evasion in solid tumor treatment, such as reduced antigenicity, decreased immunogenicity, and the development of a suppressive tumor immune microenvironment, leading to the limited killing effect of T cells on solid tumors.^[^ ^205^, ^206^ ^]^ There is evidence that the overexpression of DNA methyltransferases and immune‐suppressive adenosine is the main obstacle to T cell activation. This strategy designed a prodrug nano‐micelle with the main components of the epigenetic regulator γ‐oryzanol (Orz), adenosine inhibitor α,β‐methylene adenosine 5′‐diphosphate (AMPCP) to address the resistance to immune checkpoint therapy. Orz can upregulate the expression of GSDMD, and AMPCP can transform procaspase‐1 into active caspase‐1 by increasing ATP content, thereby leading to the cleavage of GSDMD and inducing tumor cell pyroptosis. In addition, Orz can enhance the expression of PD‐L1 and make tumors sensitive to anti‐PD‐L1 therapy. This GSH‐responsive synergistic strategy can greatly improve the therapeutic effect and inspire the design of similar drugs.^[^ ^62^ ^]^ Another example is the pro‐pyroptotic nano‐drug designed by Wang et al. based on the combined strategy of DNA methyltransferase inhibitors and X‐ray responsive ROS enhancers. The drug first restores the normal expression of functional GSDME protein by relieving the hypermethylation of tumor cells, and then X‐ray responsive W6+ can promote the production of ROS and subsequently initiate the activation of Caspase‐3 through the potential pathway of Bax‐cytoochrome C‐Caspase‐9‐Caspase‐3. Activated Caspase‐3 can cleave GSDME, which is upregulated by EGCG demethylation, leading to pore formation. The formed GSDME pores allow intracellular DAMPs to pass through and be released from the cell, stimulating the immune system and expanding the antitumor immune response.^[^ ^51^ ^]^ This strategy significantly reshapes and improves tumor immunity in traditional radiotherapy through epigenetic pyroptosis, providing new insights for clinical radiotherapy. The current clinical drugs related to pyroptosis lack specificity; for example, dimethyl fumarate, broad‐spectrum anti‐inflammatory drugs, and monoclonal antibodies targeting IL‐1β all play a role in drugs by regulating the upstream and downstream pathways of Gasdermins. Therefore, designing a drug that can specifically inhibit the function of Gasdermins has great clinical value. Since activated Gasdermins can bind to cardiolipin to form pores, Pan et al. designed a spatiotemporally selective siRNA delivery system (PMRC@siGSDME) composed of the cell membrane of pyroptotic cells, liposomes containing cardiolipin, and GSDME siRNA.^[^ ^79^ ^]^ Studies have shown that there are significant differences in protein expression patterns between normal cell membranes and pyroptotic cell membranes, thus endowing the fused cell vesicles with the ability to target pyroptosis. The results show that PMRC@siGSDME selectively targets the site of inflammation after systemic administration and shows specific accumulation within pyroptotic macrophages. This system can release GSDME siRNA through the pyroptosis reaction to suppress macrophage pyroptosis, thereby inhibiting the progression of autoimmune diseases.

Currently, it is widely recognized that utilizing cell membranes for drug delivery is a promising biological vector. Combining this with the physiological and pathological characteristics and molecular mechanisms of pyroptosis, a pyroptotic cell membrane capable of carrying nanoparticles has been designed. This approach has become a clinically valuable targeted drug delivery vector for diseases that require an enhanced immune response.

#### 4.2.3 Targeting Mitochondria

Structurally, mitochondria possess the inner mitochondrial membrane, outer mitochondrial membrane, and unique mtDNA; functionally, mitochondria can regulate the generation of ROS, cellular metabolism, ion balance, and caspase activation, which lead to programmed cell death.^[^ ^10^, ^41^, ^207^ ^]^ Excessive accumulation of ROS can trigger or exacerbate inflammatory responses by activating inflammatory signaling pathways, enhancing the activation of inflammasomes, and promoting the release of pro‐inflammatory factors. Based on the response modes of smart nanomaterials, they can be divided into microenvironmental stimulus‐response patterns and extrinsic stimulus‐response patterns. Smart nanomaterials targeting mitochondria can induce pyroptosis and achieve immune modulation by damaging the normal function of mitochondria in target cells (damage methods include redox imbalance, reduced ATP synthesis, mtDNA damage and release, ion overload, etc.).

The imbalance of mitochondrial redox can generate a large amount of ROS, which regulates mitochondrial autophagy, the formation of neutrophil extracellular traps, and the activation of NLRP3 inflammasomes.^[^ ^208^ ^]^ Inflammatory cells shift from oxidative phosphorylation to glycolysis, thereby inhibiting ATP synthesis and leading to ROS production, which promotes the progression of inflammation.^[^ ^51^ ^]^ Since the radius of action of ROS is only ≈20 nm, it is crucial to target mitochondria and effectively remove ROS near the mitochondria. Current nanomaterials mainly link ROS, redox, and mitochondrial dysfunction in an organic manner for strategic design.

**Figure 10:**
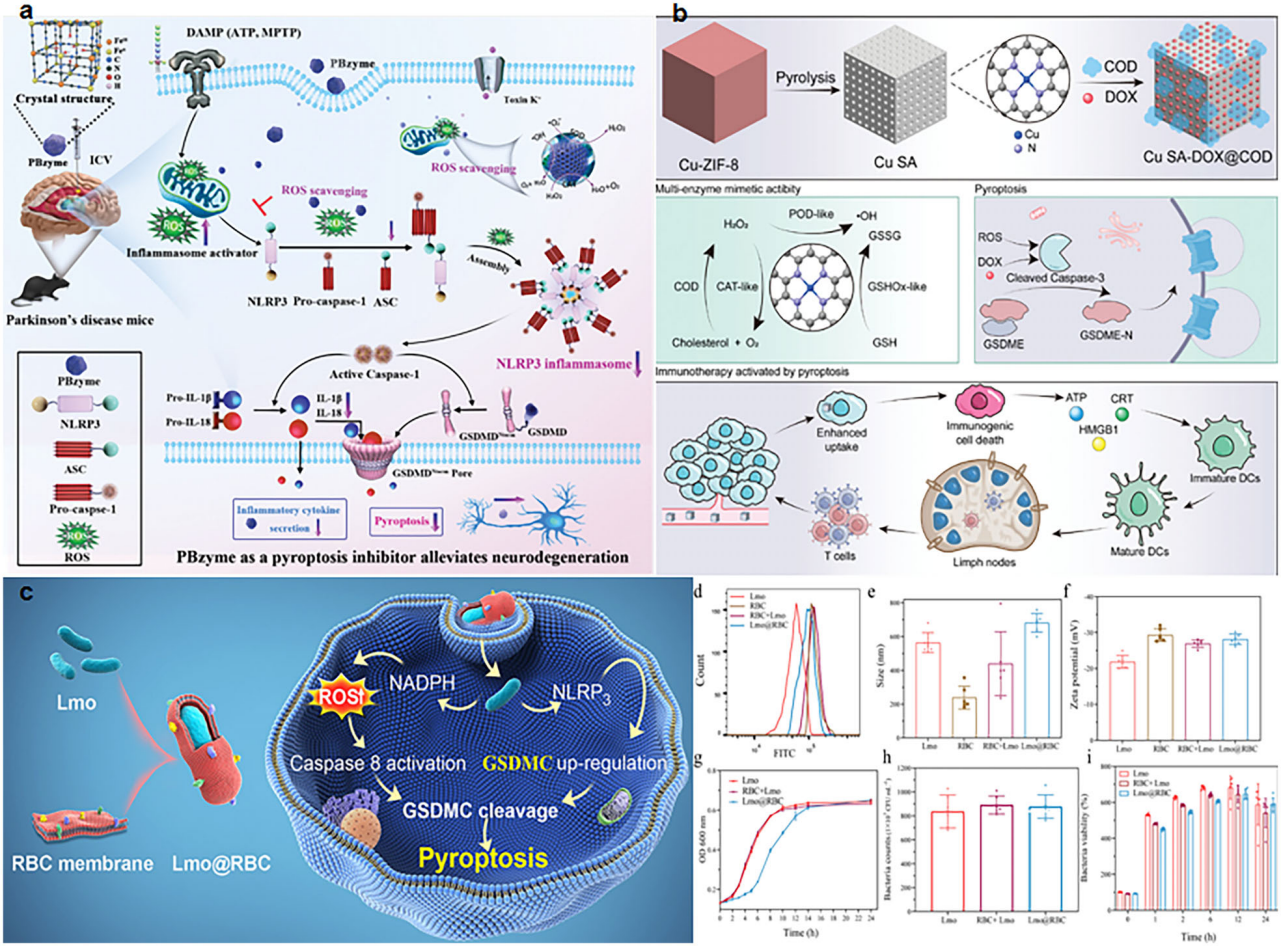
The working content of mitochondria‐targeted pyroptosis regulators. a) Schematic diagram of prussian blue nanozyme. Reproduced with permission.^[^ ^65^ ^]^ Copyright 2022, Wiley. b) Schematic diagram of Cu SA‐DOX@COD. Reproduced with permission.^[^ ^68^ ^]^ Copyright 2024, Wiley. c) Schematic diagram and characterization images of Lmo@RBC. Reproduced with permission.^[^ ^60^ ^]^ Copyright 2022, ACS.

In some molecular mechanisms of natural materials reshaping the inflammatory microenvironment, the overexpression of ROS has also been observed. Excessive ROS can cause oxidative stress in tumor tissues and indicate mitochondrial dysfunction.^[^ ^29^, ^32^, ^37^ ^]^ For instance, as shown in **Figure** **10**, Schiffelers et al. designed a dual‐responsive nano‐drug delivery system targeting the efficient delivery of paclitaxel to tumor tissues.^[^ ^209^ ^]^

Prussian blue nanoenzymes, designed based on the strategy of targeting the regulation of mitochondrial ROS, can reduce the activation of microglial NLRP3 and Caspase‐1 by scavenging ROS, thereby inhibiting the cleavage and pore formation of GSDMD to suppress the pyroptosis of microglia and alleviate the expression of neuroinflammation. This design strategy provides valuable mechanistic insights and potential therapeutic strategies for the development of nanodrugs targeting the cascade of cellular pyroptosis.^[^ ^65^ ^]^ Bo Xu et al. developed a single‐atom nanoenzyme platform (Cu SA‐DOX@COD) to activate antitumor immune responses through a GSDME‐dependent pathway. The high expression of GSH in the tumor microenvironment allows this platform to catalyze the production of ROS and consume reduced glutathione through intrinsic multienzyme‐like activities, providing oxygen sources for cholesterol (CHOL) oxidation, thereby triggering pyroptosis. The loaded CHOL and doxorubicin have synergistic effects, further enhancing pyroptosis, boosting the immunogenicity of tumor cells, and reshaping the immunosuppressive TME, thus activating systemic antitumor immune responses.^[^ ^68^ ^]^ Since most tumor antigens have strong homology with self‐proteins and are initially presented to the immune system by tumor cells without co‐stimulatory signals, they may induce immune tolerance rather than active T cell responses.^[^ ^210^ ^]^ Therefore, Yao Liu et al. designed a bacterial therapy‐based nanodrug composed of Listeria monocytogenes (LMO) selectively deficient in virulence factors, which has low immunogenicity and exhibits strong innate and adaptive immunity in many models. Unlike the mechanisms of most pyroptosis‐targeting nanodrugs, after genomic screening, it was found that the biomimetic Lmo@RBC directly kills tumor cells and induces Caspase‐8 activation by NADPH oxidase‐mediated ROS, upregulating GSDMC expression, thereby reversing the immunosuppressive tumor microenvironment and promoting systemic antitumor immune responses, effective against both solid tumors and tumor metastasis.^[^ ^60^ ^]^ These cell membrane‐coated nanoparticles not only inherit some functions of the source cells but also possess high biocompatibility, low immunogenicity, immune‐evasion capabilities, prolonged circulation time in vivo, reduced clearance by the reticuloendothelial system (RES), and excellent tumor targeting ability. There are also plans to load specific antibodies into the cell membrane to enhance its targeting of tumor cells.

Under normal physiological regulation, cardiolipin is located on the matrix side of the inner mitochondrial membrane, with only a small amount present in the outer mitochondrial membrane. The damage signature of mitochondria is the high expression of cardiolipin on the outer mitochondrial membrane, which is due to a class of PLSCR3 enzymes that flip it from the interleaflet to the outer leaflet, and then to the outer mitochondrial membrane. The binding affinity of GSDMD‐NT to cardiolipin on mitochondrial and bacterial membranes is stronger than its binding affinity to acidic phospholipids on the cell membrane. When GSDMD enters the mitochondrial bilayer membrane, it releases substances from the intermembrane space and the mitochondrial matrix, such as mtDNA. It is important to note that the release of PNPT1 leads to the degradation of total mRNA, further enhancing pyroptosis. Therefore, the design strategy for nanodrugs targeting mitochondria to induce pyroptotic reactions can also focus on increasing the exposure of cardiolipin on the mitochondria of tumor cells, thereby promoting pore formation and enhancing pyroptotic reactions, leading to an expansion of the immune response.

In summary, mitochondria, as organelles with the function of maintaining cellular homeostasis, are closely related to the regulation of inflammation and can be targeted by nanomaterials with targeting functions, such as peptides, liposomes, lipophilic cationic nanomaterials, Rhodamine, TPP, etc.^[^ ^211^, ^212^, ^213^ ^]^ These nanodrugs that target mitochondria and induce pyroptotic cascade reactions can be designed in conjunction with other aerobic strategies, drugs, or stimulus‐responsive targeting groups to enhance antitumor immune responses and anti‐inflammatory immune responses.

#### 4.2.4 Other Targeting

**Figure 11:**
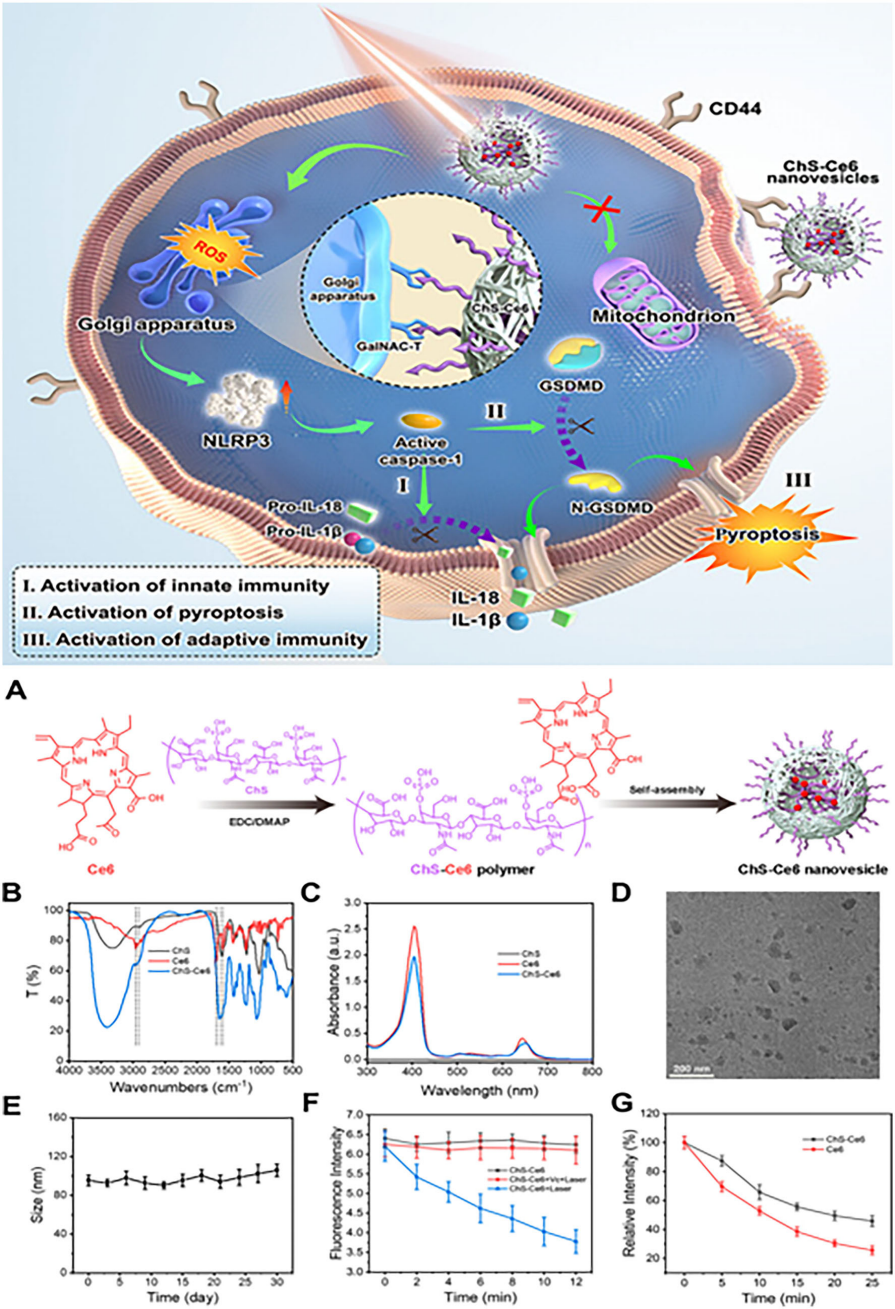
The working content of pyroptosis regulators with other regulatory strategies. Reproduced with permission.^[^ ^215^ ^]^ Copyright 2023, ACS.

Previous studies have suggested that NLRP3 is activated by translocating to the mitochondria; however, recent research has proposed a different view, namely that the recruitment, aggregation, and activation of the NLRP3 inflammasome require the Golgi apparatus rather than the mitochondria.^[^ ^214^ ^]^ Based on the aforementioned content, as shown in **Figure** **11**, Zhi‐Zhao Hu and colleagues hypothesized that the activation of the downstream signaling axis of NLRP3, the pyroptosis pathway, is associated with the Golgi apparatus. Therefore, they designed a self‐assembling nanovesicle with Golgi apparatus targeting capabilities using chondroitin sulfate (ChS) and the classic photosensitizer Ce6. ChS‐Ce6 is connected to the nanovesicle through intermolecular hydrophobic interactions, and the hydrophilic main chain on the surface of the nanovesicle enables targeting of the Golgi apparatus and tumors. This Golgi apparatus‐targeted PDT can promote the activation of NLRP3, the dependent release of caspase‐1, induce the pore formation of GSDMD, and ultimately lead to ICD in cancer cells, promoting the maturation of dendritic cells DC, increasing the infiltration of cytotoxic T lymphocytes (CTLs) in tumors, and activating long‐lasting immune memory.^[^ ^215^ ^]^ This new strategy targeting the Golgi apparatus can refer to the regulation methods of other organelles while combining the unique characteristics of the Golgi apparatus itself, bringing new insights to the development of pyroptosis‐related drugs.

As a pre‐event of Gasdermin pore formation, immune activation signal plays a triggering role in pyroptosis. The above examples show that the signal pathway of pyroptosis can be effectively activated or inhibited by regulating the expression of immune activation signal. In addition, this kind of functional material can also design various response strategies according to the characteristics of different diseases, such as acid response and GSH response in tumor tissues, which provides reference and help for the targeted regulation of diversified drug delivery systems in the future.

### 4.3 Targeted Regulation of Immune Reprogramming Following Pore Formation

#### 4.3.1 Targeted Regulation of Cytoplasmic Contents

After the formation of Gasdermins pores, cellular contents leak out of the cell and enter the body, triggering an inflammatory response. However, cellular contents are diverse, and current research has mainly focused on the markable inflammatory factors IL‐1β and IL‐18, danger signal molecules such as ATP and HMGB1, potassium ions, and some organelles and enzymes, etc.^[^ ^41^ ^]^

**Figure 12:**
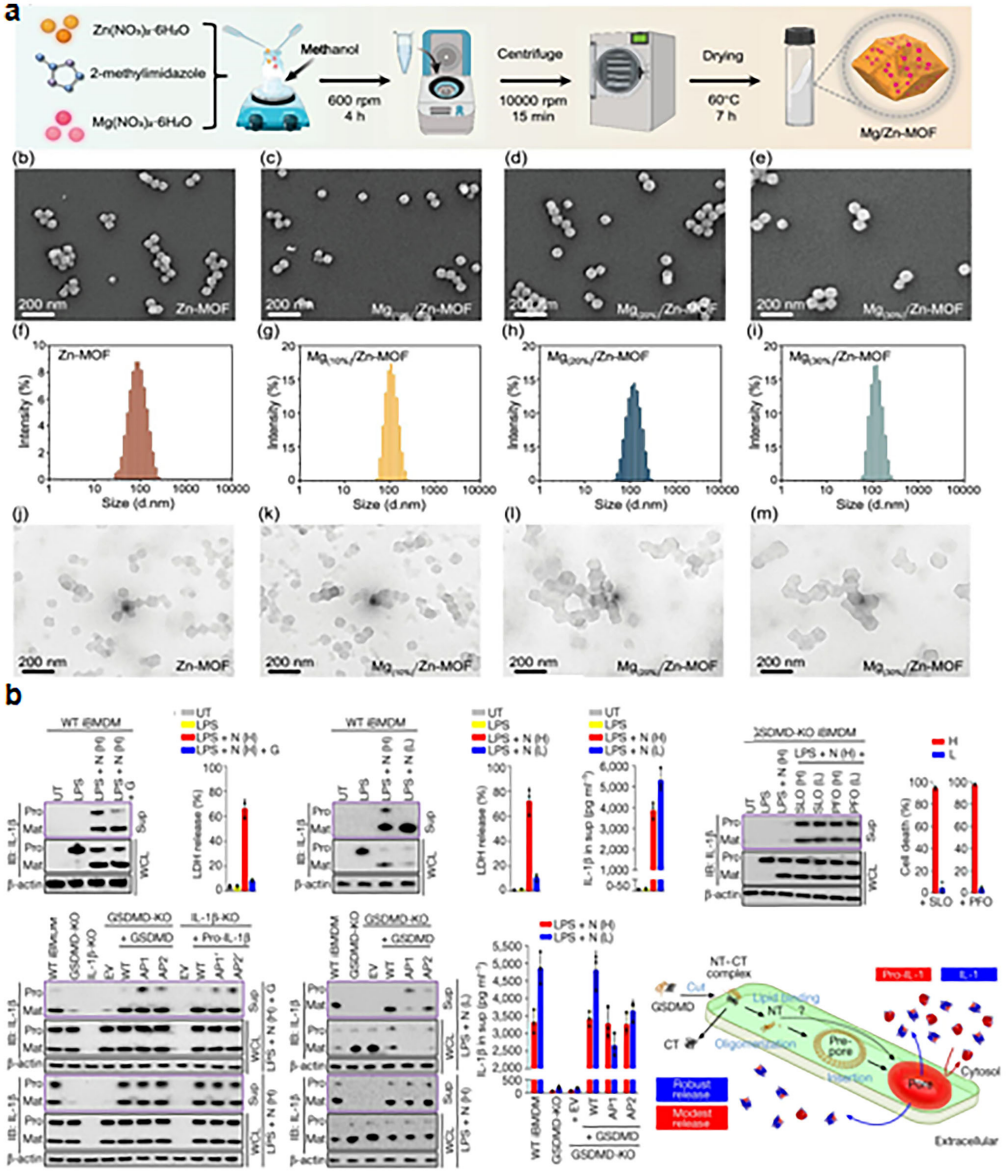
The working content of pyroptosis regulators involving inflammatory factors in design strategies. a) Schematic diagram of Mg/Zn‐MOF mechanism. Reproduced with permission.^[^ ^41^ ^]^ Copyright 2024, Oxford Academic. b) Experimental validation of preferential release of mature IL‐1β. Reproduced with permission.^[^ ^89^ ^]^ Copyright 2021, Springer Nature.

For instance, as shown in **Figure** **12**, Yang and colleagues recently designed an ATP‐responsive bimetallic metal–organic framework (MOF) based on an ion interference strategy (Mg/Zn), which uses ion interference to modulate immune responses and prevent tissue damage. Magnesium and zinc ions released from the framework synergistically inhibit the formation of membrane pores by reducing the expression and activation of GSDMD. Mechanistically, this is mainly achieved by interfering with the NLRP3/Caspase‐1/GSDMD classical inflammasome signaling pathway and the Caspase‐11/GSDMD non‐classical inflammasome signaling pathway. This approach addresses the challenges of synergistic ion effects and targeted delivery faced by traditional immune‐modulating nanomaterials, providing inspiration for the design of responsive pyroptosis nanomaterials. In subsequent nanomaterial designs, materials corresponding to pyroptosis‐related cellular contents can also be considered to achieve precise drug release capabilities.^[^ ^41^ ^]^

Furthermore, as mentioned earlier, recent studies have shown that by regulating the expression levels of GSDMD‐NT, the size of the pores in pyroptotic cells can be controlled. In other words, the excretion of cellular contents can be finely controlled by precisely regulating the size of the pores. For example, if organelle excretion is not desired, the expression of GSDMD‐NT can be reduced to form only small pores. However, it should be noted that since GSDMD pores are mainly negatively charged, while pro‐IL‐1β is also negatively charged, IL‐1β is positively charged. Therefore, pro‐IL‐1β is less likely to pass through the pores due to electrostatic repulsion and will be retained within pyroptotic cells.^[^ ^89^ ^]^

#### 4.3.2 Targeted Modulation to Disrupt Immunological Microenvironment

Natural functional materials have been discovered to modulate the expression of gasdermins for cancer treatment. Additionally, a variety of natural materials targeting gasdermins are currently used for the treatment of inflammatory diseases, such as birch tar acid for osteoarthritis, Xiaoqinglong soup for allergic rhinitis, and Xianglian pills for ulcerative colitis.^[^ ^22^, ^23^, ^33^ ^]^ Some natural materials targeting gasdermins enhance the immune response by inducing pyroptosis to achieve anti‐inflammatory effects,^[^ ^24^, ^29^, ^31^ ^]^ while others inhibit pyroptosis to prevent the production of inflammatory factors, and some have mixed effects.^[^ ^21^, ^26^, ^27^ ^]^ As shown in **Table** **1**, extracts of Ganoderma lucidum and Cordyceps militaris can enhance the immune response by regulating the occurrence and development of pyroptosis.^[^ ^29^, ^37^ ^]^ Some of the natural materials listed in the table have effects on cancer that are not limited to the phenotype of pyroptosis; they can also induce apoptosis, immune responses, and other phenotypes, reshaping the tumor immune microenvironment from multiple aspects.^[^ ^35^ ^]^

**Table 1:** The impacts of natural functional materials on cancer and inflammatory diseases: molecular mechanisms and phenotypic summaries.

Number	Natural functional materials	Molecular mechanisms	Disease	Phenotype	Reference
1	TSD	NLRP3, caspase‐l, caspase‐l, pro‐caspase‐1, GSDMD, GSDMD‐N, 1L‐18, 1L‐1β	Allergic rhinitis (AR)	Pyroptosis, apoptosis, autophagy	[21]
2	XQLD	IL‐4.IL‐5.IL‐13, GSDMD, IL‐1B, IL‐18, NLRP3	Osteoarthritis (OA)	Pyroptosis	[22]
3	BA	IL‐1B.caspase‐1, GSDMD‐N, NLRP3	Cerebral ischemia‐reperfusion Injury	Pyroptosis	[23]
4	JDHX	caspase‐1 p10, GSDMD	Macrophage M1 Phenotype (M1)	Macrophage polarization、 Pyroptosis	[24]
5	XBXD	caspase‐9/3, GSDME	Chemotherapy‐induced nausea and vomiting (CINV)	Pyroptosis	[25]
6	CQCQD	GSDMD‐N, NLRP3, caspase‐1	Acute pancreatitis (AP)	Pyroptosis	[26]
7	Punicalagin	NLRP3, caspase‐1, GSDMD, GSDMD‐N IL‐1βmRNA, IL‐18 mRNA	Acute myocardial infarction (AMI) ventricular remodeling (VR)	Pyroptosis	[27]
8	Irisin	GSDMD‐N, caspase‐1, IL‐1β, IL‐18	Type 2 Diabetes Mellitus (T2DM)	Pyroptosis	[28]
9	GLE	Caspase‐3.GSDME, ROS	Breast cancer	Pyroptosis, tumor immune response, disruption of tumor adhesion, migration, invasion, colonization, and angiogenesis.	[29]
10	Aa‐ME	Caspase‐11, GSDMD	Sepsis	Pyroptosis	[30]
11	Dracocephalum moldavica	IL‐18.IL‐1β caspase‐1.GSDMD, NLRP3	Pulmonary fibrosis	Pyroptosis	[31]
12	TXL	caspase‐1, GSDMD, ROS, COX2, iNos	Pyroptosis‐associated atherosclerosis (AR)	Pyroptosis	[32]
13	XLP	IL‐1B, IL‐6, TNF‐α, IL‐18, GSDMD‐N, TLR4 NLRP3, active‐caspase‐1, MyD88. p‐NF‐KB/NF‐KB	Ulcerative colitis (UC)	Pyroptosis	[33]
14	Garcinia cambogia extract	GSDND, IL‐10, MCP‐1	Wound healing	Pyroptosis	[34]
15	CM	Caspase‐3, GSDME, PARP, GSDME‐N.	Lung cancer	Apoptosis, pyroptosis	[35]
16	Curcumin	GSDMD‐N, caspase‐1, NLRP3, IL‐1B, ILL‐18	Leukodystrophy After Ischemic Stroke	Pyroptosis	[36]
17	Curcumin	GSDME‐N, ROS, LDH	Hepatocellular Carcinoma (HCC)	Pyroptosis	[37]
18	KJL	NLRP3, caspase‐1, GSDMD‐N, IL‐1β, IL‐18	Ulcerative Colitis (UC)	Pyroptosis	[219]
19	Quercetin	NLRP3, caspase‐1, GSDMD‐N, IL‐1β	Neurodegenerative diseases	Mitophagy	[38]
20	Betaine	ROS, NLRP3, ASC, caspase‐1, GSDMD, GSDMD‐N, IL‐18, IL‐1β, LDH	Dementia	Pyroptosis	[39]
21	Quercetin	NLRP3, AIM2, caspase‐8, GSDMD	Hepatic ischemia‐reperfusion injury	Pyroptosis	[40]

**Figure 13:**
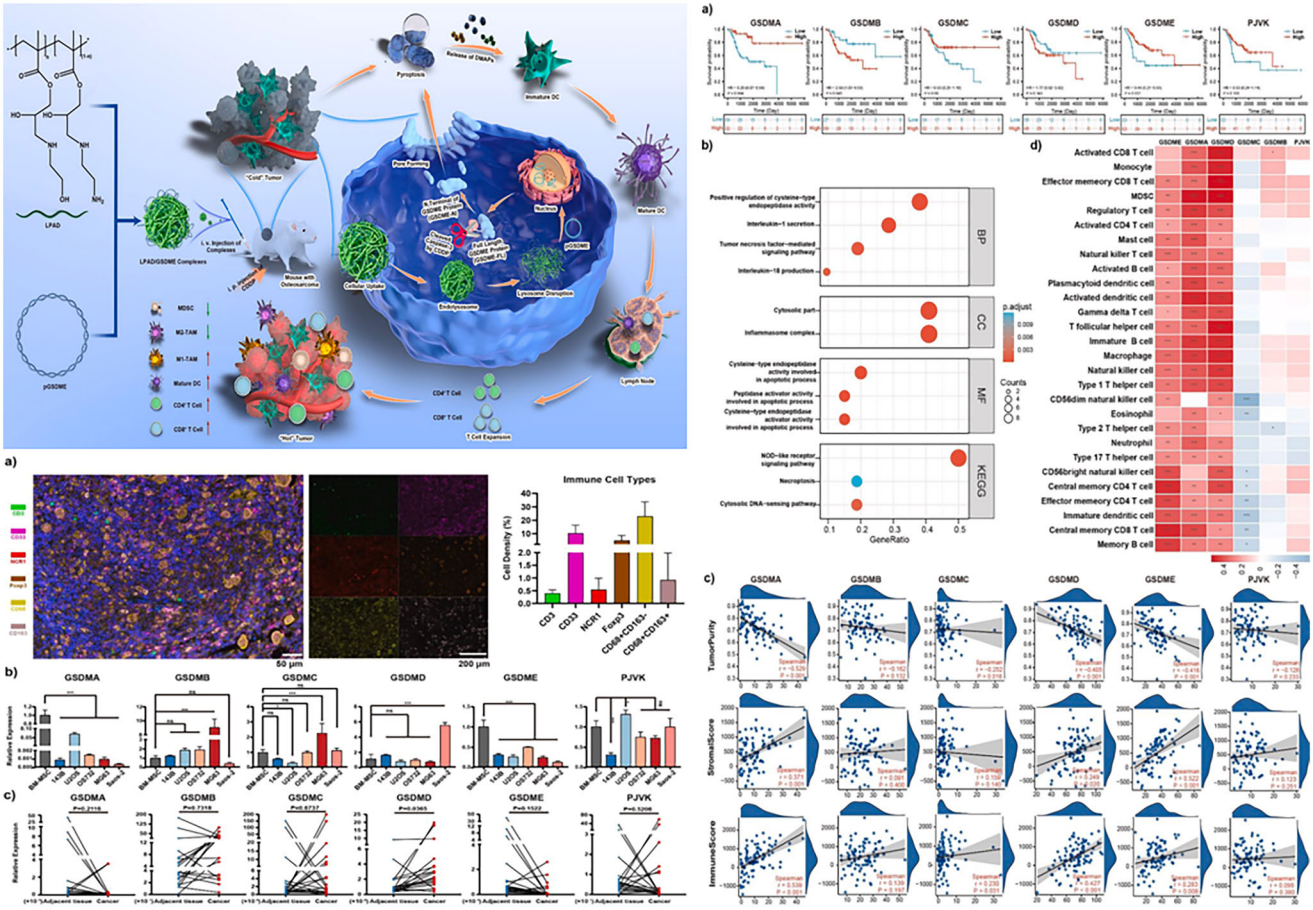
Pyroptosis inducers that aid in reshaping the immune microenvironment. Reproduced with permission.^[^ ^216^ ^]^ Copyright 2024, Elsevier.

As shown in **Figure** **13**, Nie et al. found that the expression of GSDME is positively correlated with the prognosis and immune infiltration of osteosarcoma patients. To address the highly immunosuppressive tumor microenvironment (TME) of osteosarcoma, they designed a carrier rich in hydroxyl groups for the formation of a hydration layer to deliver the GSDME gene, thereby enhancing the degree of pyroptosis induced by cisplatin. These materials can initiate the release of pro‐inflammatory cytokines through pyroptosis, increase immune cell infiltration, activate adaptive immune responses, and create a favorable immunogenic hot TME, converting “cold tumors” to “hot tumors.” This study not only confirmed the role of GSDME in immune infiltration and prognosis in osteosarcoma but also provided a reference for the design strategy of biomaterials reshaping the tumor microenvironment (**Table** **2**).^[^ ^216^ ^]^

**Table 2:** Summary of the impact of intelligent composite functional materials on cancer and inflammatory diseases, along with their molecular mechanisms and phenotypes.

Name	Main components	Type	Main targets	Strategies	Cell lines	Synergy methods	References	Response
/	Mg/Zn‐MOF	MOF	GSDMD	Inhibition of pore formation	L929	/	[41]	ATP
RpMPs	Melanin	/	GSDMD	Canonical inflammasome pathway	RAW 264.7;HUVEC	Fluorescence imaging	[42]	/
TPN	EGCG, Mn	Nanoparticles	GSDMD	Anti‐inflammatory effect	RAW264.7, HUVECs, HEK293T;THP‐1; Primary peritoneal macrophages	/	[43]	/
Pt‐NS/HCS	Pt	Nanozymes	GSDMD GSDMD	POD‐like activity	CT26	PD‐L1	[44]	/
CTEP	CM, TK membrane	Nanosheets	Membrane anchoring	Catalase mimetic activity	Hepa1‐6	PDT, PD‐L1		H2O2/PH
CSSG	Ca2+, Nd3+, DPTU	/	GSDME, GSDMD	/	HeLa	NIR‐II fluorescence imaging	[45]	/
(Nig + DAC)@HmA	Nig, DAC	/	GSDMD	/	MB49 RAW264.7 4T1	/	[46]	/
LPAD/GSDME Complexes	LPAD/GSDME, CDDP	Gene Delivery System	GSDME	ICD	K7M2	Combination chemotherapy drugs	[47]	/
ACNPs	5‐Aza, oHSV	Nanoprodrug	GSDME	/	4T1	Synergy combination	[48]	ROS;PH
4‐OI/BLipo	4‐OI	Liposome	GSDME	/	NCM460, IEC‐6	/	[49]	/
DMP@P	DCT, MIT	Mesoporous Polydopamine	GSDME	Epigenetics	4T1	PDT	[50]	PH
PWE	EGCG, W6+	Metal‐Phenolic Networks	GSDME, Mitochondria	Epigenetics	4T1	X‐RAY	[51]	/
SALDT	DCT, Triclabendazole, DSPE‐PEG2000‐SA	Liposome	GSDME	ICB	4T1	ICB ICD, PD‐1	[52]	/
HfO2NPs	DAC	NPs	ROS, GSDME	Epigenetics	TNBC	Switching from Apoptosis to Pyroptosis	[53]	X‐ray
	DAC, TBE	/	STING, GSDME	/	4T1	PDT, NIR fluorescence imaging	[54]	ROS
IDN@MC	Macrophages, DAC	Biohybrid microrobots	Tumor, GSDME	/	4T1	Photothermal conversion, Fluorescence navigation	[55]	pH
Nano‐CD	sgRNA, Cisplatin	/	GSDME	/	B16F10, A375, 293T	Immune checkpoint blockade	[56]	PH
TSD@LSN‐D	DAC, PD‐L1 blocking peptide, TPRA	/	GSDME, PD‐L1	Switching from Apoptosis to Pyroptosis	RM‐1	PDT	[57]	pH
PNM	PI3K/mTOR inhibitor, Flav		GSDME	Reprogramming the immunosuppressive tumor microenvironment	4T1	PD‐1	[58]	GSH
DOX/JQ1 ‐IBRN	DOX, JQ1	Implantable nanoarrays	GSDME	/	4T1	PD‐L1	[59]	ROS
Lmo@RBC	Listeria monocytogenes with selective deletion of virulence factors, Red blood cell membrane	Biomaterials	ROS, GSDMC	lower systemic inflammatory response	CT26	/	[60]	/
Cu‐Pic/HA NPs	Cu, Pic, HA	NPs	Polyamines	mitochondrial dysfunction, Epigenetics	4T1	Cuproptosis, SOD like, CAT like, POD like	[61]	/
AOZN	Orz、AMPCP	Prodrug nano‐micelles	GSDMD, ATP	Epigenetics	B16F10, CT26	PD‐L1	[62]	GSH
CCNP	Camptothecin	Nanoparticles	ROS, mtdna	/	4T1	αPD‐1, pdt	[63]	ROS/GSH
/	Iridium(III)‐triphenylamine	Photosensitizer	DNA	AIM2	/	Ferroptosis	[64]	/
MPTP	Prussian Blue	Artificial nanozymes	ROS	Pyroptosis	BV2	/	[65]	/
hCZAG	Cu 2+, Zn 2+	ZIF‐8	ROS	ICD	4T1	Photothermal effect, cascade catalytic activity, and GSH depletion capability, enabling image‐guided tumor therapy, Fenton	[66]	/
(PG@Cu‐FP	EGCG, Cu 2+	Metal‐phenolic networks	Mitochondria	Proton Sponge Effect, Lysosomal Escape Capture	Primary rat nucleus pulposus tissue	/	[67]	/
Cu SA‐DOX@COD	Cu, DOX, COD	ZIF‐8Nanozymes	ROS	Depletion of reduced glutathione (GSH) in tumor cells, Alleviating hypoxia	RM‐1	CAT‐like;POD‐like;GSHOx‐like	[68]	/
nab‐TTVPHE	HSA, TTVPHE	AIE PS	ROS, Mitochondria	/	A375, QBC939	PDT	[69]	/
/	NBPy	Photocatalyst	Mitochondria, ROS	/	Hela, 4 T1	ICD; PDT	[70]	/
/	Th‐M	AIE	Mitochondria	/	CAL27	PDT	[71]	/
HCS‐FeCu	Fe, Cu	Hollow carbon nanozymes	ROS	PD‐1, ICB	4T1	Multiple enzyme‐mimicking activities	[72]	Photoresponsive
CaNMs	Ca 2+, CUR	/	ROS	/	4T1	/	[73]	PH
/	NI‐TA	AIE	ROS	/	T47D	PDT	[74]	/
/	Cu、Fe and Ni	COF	OH	/	4T1	SOD‐like, POD ‐like, GPx‐like, CDT, PTT	[75]	/
MCPP	PTX, P18	Nanoprodrugs	Mitochondria	/	CT26	PDT	[76]	ROS/GSH
AFRM	C60, β‐Ala	Redox modulators	GSDMD	Regulation of macrophage oxidation, inhibition of excessive inflammation activation	RAW 264.7; IEC‐6; HUVEC	SOD‐like;POD‐like;	[77]	
/	EGCG, Mn	Carrier‐free dual‐functional nanoinhibitors	Caspase‐1	Canonical inflammasome pathway	HCT116; GSE1; HMrSV5; MGC803; HGC‐27; CT26	/	[78]	/
PMRC@siGSDME	Pyroptotic macrophage membranes, R8‐cardiolipin‐containing nanovesicles, siGSDME	Pyroptosis cell membrane hybrid nanovesicles	Inflammatory tissue	Spatio–temporal selective inhibition	ATDC5, RAW 264.7	/	[79]	/
/	Silylene, Mesoporous silica	Nanosheets	Caspase‐3	/	A375	PTT	[80]	pH/Thermoresponsive

After the formation of the Gasdermin pore, the cells burst and released the cell contents, among which inflammatory factors such as IL‐1β, IL‐18, and ATP mainly mediated the post‐pore event, namely immune reprogramming. At present, the design of these functional materials mainly focuses on the events preceding pore formation and the pore formation process itself, with limited research dedicated to immune reprogramming within the body. However, strengthening the research in this area is of great significance for developing functional materials for the treatment of inflammatory diseases.

## 5 Prospects on the Advantages and Challenges of Biomaterials for Targeting Gasdermin Family Regulation: From Research and Development to Clinical Translation

In recent years, researchers have been continuously developing carriers loaded with pyroptosis activators or inhibitors and functional materials with pyroptosis‐regulating capabilities. This article categorizes targeting strategies into direct targeting of the Gasdermins family for direct regulation, transcription, and epigenetic regulation strategies, strategies for converting “cold tumors” into “hot tumors,” functional materials with artificial nanoenzyme synergistic function strategies, and other strategies.

Functional materials that directly target the Gasdermins family have the advantages of simple design and convenient synthesis. However, there are also risks of over‐activation, weak targeting, and selectivity. Due to the promoter methylation of the GSDME gene, the expression level of GSDME in tumor cells is much lower than in normal cells. Therefore, most of the designs for transcription and epigenetic regulation strategies target the protein GSDME and are generally designed as multi‐drug synergistic nano‐platforms. That is, the target protein expression is upregulated first, and then pyroptosis is induced by pyroptosis inducers. These platforms have the advantages of expanding drug efficacy and synergistic effects. When used in combination with PD‐L1 therapy, they can convert “cold tumors” into “hot tumors,” combating the resistance to ICD treatment. However, the actual clinical application value and the stability of the drugs during storage of the nano‐platforms deserve attention. Due to the high characteristic of GSH in tumor tissues, functional materials that target the inflammasome and mitochondria to induce pyroptosis cascade reactions can choose to increase ROS expression as the core event. By increasing ROS expression, the pyroptosis cascade reaction is accelerated, leading to the depletion of GSH within the tissue and subsequently activating the immune system.

The most widely applied bioactivity of the Gasdermins family is its targeting of the plasma membrane pore‐forming activity. Currently, most articles are designed and synthesized based on this. However, studies have shown that the Gasdermins family can form pores not only in the plasma membrane but also in cellular organelles. For example, GSDMD can target the nuclear membrane in neutrophils, promoting its breakdown and NET formation. It has also been proven to target mitochondria, with the N‐terminus able to bind to a type of acidic lipid on the inner mitochondrial membrane, namely cardiolipin. Similar studies have been published on other Gasdermins. In subsequent research, similar schemes can be considered to induce damage to tumor tissue organelles to play an antitumor role.^[^ ^118^, ^217^ ^]^

Natural materials originate from plants, animals, and microorganisms, and their safety has been clinically verified. A large part of the current research on drugs also has the characteristic of being both food and medicine, so they have the potential to prepare long‐term medication. However, due to the numerous components of natural materials, their drug targets are also numerous, making the screening and characterization of target components and mechanisms of natural materials one of the difficulties for researchers. At the same time, because some natural drugs are produced in small quantities, further research is needed on the extraction and synthesis of effective components. There are also a few studies on natural material targeting drug delivery systems published. This drug delivery system can not only improve the bioavailability of natural materials but also design different response modes according to different disease characteristics, which is more efficient and targeted. In summary, natural materials have the characteristics of multiple targets and high safety, but deeper research is still needed to overcome the problems of target component and mechanism screening and characterization, and effective component extraction and synthesis, to fully utilize the therapeutic potential of natural materials in reshaping the inflammatory microenvironment.

Functional material platforms in this field often carry drugs that respond to light, ionizing radiation, and X‐rays, and some materials also have synergistic imaging systems to assist precision medicine, providing a possible solution to prevent excessive inflammation mentioned earlier. The timeline of different cell death methods in diseases is not arranged longitudinally but horizontally. Therefore, using strategies that synergistically treat with various cell death methods, such as apoptosis, ferroptosis, and cuproptosis, is feasible, which can avoid the problems of single‐mode treatment, such as “drug resistance.” However, it should be noted that it should not produce harmful off‐target effects and increase the risk of other diseases. This is an important consideration for the safety and effectiveness of treatment. In addition, functional material platforms can be endowed with pH‐responsive, GSH‐responsive, thermosensitive, photothermal‐responsive, and ATP‐responsive groups, or by coating cell membranes, exosomes, loading artificial nanoenzymes, and other methods to improve their targeting and selectivity, cell uptake, antigen presentation, and T cell uptake and activation capabilities, and other immune‐related abilities.

**Figure 14:**
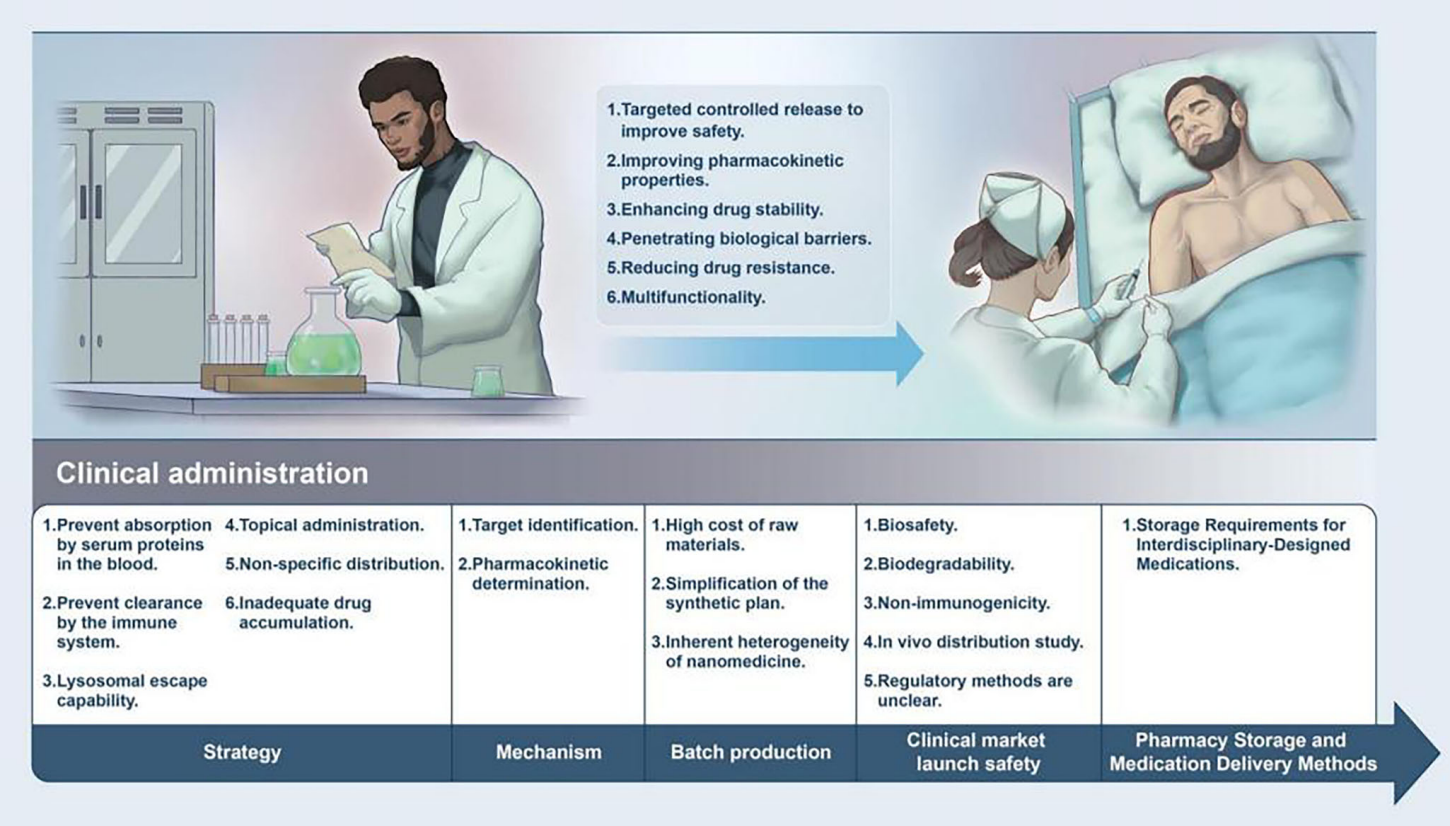
Design issues of gasdermin‐targeting nanomedicines from synthesis to clinical translation.

Moreover, as shown in **Figure** **14**, due to the first‐pass effect of nanomaterials in the liver after entering the bloodstream, they mainly accumulate in the liver tissue, reducing the effective utilization rate of drugs. For other suitable diseases, in situ injection hydrogels can also be used for direct drug administration to improve targeting. After nanodrugs enter the body, they may be quickly cleared by endosomes, so drug design should pay attention to the endosome escape ability of the material.

Furthermore, multifunctional drug delivery system combined with pyroptosis has become a key area of research for the treatment of cancer and inflammatory diseases. However, translating these functional materials into clinical applications still requires ongoing exploration. Therefore, future research should focus on solving the following promising research problems and exploring other possibilities. First of all, personalized diagnosis and treatment plan should be developed based on the individual characteristics of patients (such as genes, tumor microenvironment) and the stage of illness; Second, accelerating the process of clinical transformation of these functional materials should be prioritized; Thirdly, although the mechanism of Gasdermin family and pyroptosis in some cancers is deepening, their roles in some inflammatory diseases still needs further investigation; Finally, a single treatment plan and research method often lead to drug resistance, so the future drug design can consider multidisciplinary cooperation and multi‐method joint application. In recent years, the quantum computer, as a technology that can quickly process large‐scale data, has deserved attention. It can significantly accelerate the modeling of interactions between a large number of compounds and molecules, help drug discovery, and analyze complex health data sets. This means that after the target is confirmed, the screening of candidate compounds can be accelerated, the drug development cycle can be greatly shortened, and the research efficiency can be improved. However, at present, the hardware of the quantum computer is not fully mature, and more advanced equipment is needed to support complex research tasks.^[^ ^218^ ^]^ At the same time, CRISPR technology is gradually maturing. Future research can focus on developing gene editing nanocarriers and regulating the expression of the Gasdermin gene so as to achieve a fundamental treatment plan and bring more lasting therapeutic effects. Nevertheless, this direction still needs to address the off‐target effects and ethical concerns to ensure the safety and ethicality of the treatment.

In short, long‐term toxicity studies of drugs acting on animal models in this field are rarely conducted, but safety testing is a prerequisite for high druggability. Subsequent research can also focus more on preclinical and clinical trials to evaluate the therapeutic effects, long‐term safety, and in vivo toxicity of pyroptosis‐inducing nanomaterials and continuously promote the clinical translation of drugs. It is worth mentioning that the development of organoids in recent years has brought new vitality to toxicity and pharmacodynamics testing, and the intersection of nanofunctional materials and organoids may accelerate the clinical translation speed of nanodrugs.

## Conflict of Interest

The authors declare no conflict of interest.

## Author Contributions

L.T., and S.P. contributed equally to this work.
